# Common Genetic Variants and Modification of Penetrance of *BRCA2*-Associated Breast Cancer

**DOI:** 10.1371/journal.pgen.1001183

**Published:** 2010-10-28

**Authors:** Mia M. Gaudet, Tomas Kirchhoff, Todd Green, Joseph Vijai, Joshua M. Korn, Candace Guiducci, Ayellet V. Segrè, Kate McGee, Lesley McGuffog, Christiana Kartsonaki, Jonathan Morrison, Sue Healey, Olga M. Sinilnikova, Dominique Stoppa-Lyonnet, Sylvie Mazoyer, Marion Gauthier-Villars, Hagay Sobol, Michel Longy, Marc Frenay, GEMO Study Collaborators, Frans B. L. Hogervorst, Matti A. Rookus, J. Margriet Collée, Nicoline Hoogerbrugge, Kees E. P. van Roozendaal, HEBON Study Collaborators, Marion Piedmonte, Wendy Rubinstein, Stacy Nerenstone, Linda Van Le, Stephanie V. Blank, Trinidad Caldés, Miguel de la Hoya, Heli Nevanlinna, Kristiina Aittomäki, Conxi Lazaro, Ignacio Blanco, Adalgeir Arason, Oskar T. Johannsson, Rosa B. Barkardottir, Peter Devilee, Olofunmilayo I. Olopade, Susan L. Neuhausen, Xianshu Wang, Zachary S. Fredericksen, Paolo Peterlongo, Siranoush Manoukian, Monica Barile, Alessandra Viel, Paolo Radice, Catherine M. Phelan, Steven Narod, Gad Rennert, Flavio Lejbkowicz, Anath Flugelman, Irene L. Andrulis, Gord Glendon, Hilmi Ozcelik, OCGN, Amanda E. Toland, Marco Montagna, Emma D'Andrea, Eitan Friedman, Yael Laitman, Ake Borg, Mary Beattie, Susan J. Ramus, Susan M. Domchek, Katherine L. Nathanson, Tim Rebbeck, Amanda B. Spurdle, Xiaoqing Chen, Helene Holland, kConFab, Esther M. John, John L. Hopper, Saundra S. Buys, Mary B. Daly, Melissa C. Southey, Mary Beth Terry, Nadine Tung, Thomas V. Overeem Hansen, Finn C. Nielsen, Mark I. Greene, Phuong L. Mai, Ana Osorio, Mercedes Durán, Raquel Andres, Javier Benítez, Jeffrey N. Weitzel, Judy Garber, Ute Hamann, Susan Peock, Margaret Cook, Clare Oliver, Debra Frost, Radka Platte, D. Gareth Evans, Fiona Lalloo, Ros Eeles, Louise Izatt, Lisa Walker, Jacqueline Eason, Julian Barwell, Andrew K. Godwin, Rita K. Schmutzler, Barbara Wappenschmidt, Stefanie Engert, Norbert Arnold, Dorothea Gadzicki, Michael Dean, Bert Gold, Robert J. Klein, Fergus J. Couch, Georgia Chenevix-Trench, Douglas F. Easton, Mark J. Daly, Antonis C. Antoniou, David M. Altshuler, Kenneth Offit

**Affiliations:** 1 Department of Epidemiology and Population Health and Department of Obstetrics and Gynecology and Women's Health, Albert Einstein College of Medicine, New York, New York, United States of America; 2 Clinical Genetics Service, Department of Medicine, Sloan-Kettering Institute, Memorial Sloan-Kettering Cancer Center, New York, New York, United States of America; 3 Cancer Biology and Genetics Program, Sloan-Kettering Institute, Memorial Sloan-Kettering Cancer Center, New York, New York, United States of America; 4 Broad Institute of Harvard and Massachusetts Institute of Technology, Harvard Medical School, Boston, Massachusetts, United States of America; 5 Program in Medical and Population Genetics, Broad Institute of Harvard and Massachusetts Institute of Technology, Cambridge, Massachusetts, United States of America; 6 Center for Human Genetic Research, Massachusetts General Hospital, Boston, Massachusetts, United States of America; 7 Department of Molecular Biology, Massachusetts General Hospital, Boston, Massachusetts, United States of America; 8 Center for Cancer Research, Cancer Inflammation Program, Human Genetics Section, National Cancer Institute – Frederick, Frederick, Maryland, United States of America; 9 Centre for Cancer Genetic Epidemiology, Department of Public Health and Primary Care, University of Cambridge, Cambridge, United Kingdom; 10 Unité Mixte de Génétique Constitutionnelle des Cancers Fréquents, Centre Hospitalier Universitaire de Lyon/Centre Léon Bérard, Lyon, France; 11 Equipe labellisée LIGUE 2008, UMR5201 CNRS, Centre Léon Bérard, Université de Lyon, Lyon, France; 12 Institut Curie, Service de Génétique, INSERM U830, F-75248, Université Paris Descartes, Paris, France; 13 Service de Genetique Oncologique, Institut Curie, Paris, France; 14 Département Oncologie génétique, Prévention et Dépistage, INSERM CIC-P9502, Institut Paoli-Calmettes/Université d'Aix-Marseille II, Marseille, France; 15 Institut Bergonié, Bordeaux, France; 16 Centre Antoine Lacassagne, Nice, France; 17 GEMO Study - Cancer Genetics Network “Groupe Génétique et Cancer”, Fédération Nationale des Centres de Lutte Contre le Cancer, Paris, France; 18 Family Cancer Clinic, Netherlands Cancer Institute, Amsterdam, The Netherlands; 19 Department of Epidemiology, Netherlands Cancer Institute, Amsterdam, The Netherlands; 20 Department of Medical Oncology, Rotterdam Family Cancer Clinic, Erasmus University Medical Center, Rotterdam, The Netherlands; 21 Department of Human Genetics, Radboud University Nijmegen Medical Centre, Nijmegen, The Netherlands; 22 Department of Clinical Genetics, University Medical Center, Maastricht, The Netherlands; 23 Gynecologic Oncology Group Statistical and Data Center, Roswell Park Cancer Institute, Buffalo, New York, United States of America; 24 NorthShore University Health System, Evanston, Illinois, United States of America; 25 Central Connecticut Cancer Consortium, Hartford Hospital, Hartford, Connecticut, United States of America; 26 University of North Carolina, Chapel Hill, North Carolina, United States of America; 27 New York University School of Medicine, New York, New York, United States of America; 28 Molecular Oncology Laboratory, Hospital Clinico San Carlos, Madrid, Spain; 29 Department of Obstetrics and Gynecology, Helsinki University Central Hospital, Helsinki, Finland; 30 Department of Clinical Genetics, Helsinki University Central Hospital, Helsinki, Finland; 31 Hereditary Cancer Program, Catalan Institute of Oncology, Barcelona, Spain; 32 Department of Oncology, Landspitali–LSH, Reykjavik, Iceland; 33 Department of Pathology, Landspitali–LSH, Reykjavik, Iceland; 34 Faculty of Medicine, University of Iceland, Reykjavik, Iceland; 35 Department of Human Genetics and Department of Pathology, Leiden University Medical Center, Leiden, The Netherlands; 36 Center for Clinical Cancer Genetics and Global Health, Department of Medicine, University of Chicago Medical Center, Chicago, Illinois, United States of America; 37 Department of Population Sciences, the Beckman Research Institute of the City of Hope, Duarte, California, United States of America; 38 Department of Laboratory Medicine and Pathology, Mayo Clinic, Rochester, Minnesota, United States of America; 39 Department of Health Sciences Research, Mayo Clinic, Rochester, Minnesota, United States of America; 40 Unit of Genetic Susceptibility to Cancer, Department of Experimental Oncology and Molecular Medicine, Fondazione IRCCS Istituto Nazionale Tumori (INT), Milan, Italy; 41 IFOM, Fondazione Istituto FIRC di Oncologia Molecolare, Milan, Italy; 42 Unit of Medical Genetics, Department of Preventive and Predictive Medicine, Fondazione IRCCS Istituto Nazionale dei Tumori (INT), Milan, Italy; 43 Division of Cancer Prevention and Genetics, Istituto Europeo di Oncologia (IEO), Milan, Italy; 44 Division of Experimental Oncology 1, Centro di Riferimento Oncologico (CRO), IRCCS, Aviano (PN), Italy; 45 Moffitt Cancer Center, Tampa, Florida, United States of America; 46 Women's College Research Institute, Toronto, Canada; 47 CHS National Cancer Control Center and Department of Community Medicine and Epidemiology, Carmel Medical Center, Haifa, Israel; 48 Samuel Lunenfeld Research Institute, Mount Sinai Hospital, Toronto, Canada; 49 Cancer Care Ontario, Ontario Cancer Genetics Network, University of Toronto, Toronto, Canada; 50 Departments of Molecular Virology, Immunology, and Medical Genetics and Internal Medicine, Ohio State University, Columbus, Ohio, United States of America; 51 Immunology and Molecular Oncology Unit, Istituto Oncologico Veneto, IRCCS, Padua, Italy; 52 Department of Oncology and Surgical Sciences, University of Padua, Padua, Italy; 53 The Susan Levy Gertner Oncogenetics Unit, Institute of Genetics, Sheba Medical Center, Tel Hashomer, Israel; 54 Department of Oncology, Lund University, Lund, Sweden; 55 Division of General Internal Medicine, Department of Medicine, University of California San Francisco, San Francisco, California, United States of America; 56 Gynaecological Oncology Unit, UCL EGA Institute for Women's Health, University College London, United Kingdom; 57 Department of Oncology, The Hospital of the University of Pennsylvania, Philadelphia, Pennsylvania, United States of America; 58 Department of Cell and Molecular Biology, University of Pennsylvania School of Medicine, Philadelphia, Pennsylvania, United States of America; 59 Center for Clinical Epidemiology and Biostatistics, Department of Biostatistics and Epidemiology, The University of Pennsylvania School of Medicine, Philadelphia, Pennsylvania, United States of America; 60 Genetics and Population Health Division, Queensland Institute of Medical Research, Brisbane, Australia; 61 Peter MacCallum Cancer Centre, Melbourne, Australia; 62 Cancer Prevention Institute of California, Fremont, California, United States of America; 63 Centre for Genetic Epidemiology, University of Melbourne, Melbourne, Australia; 64 Huntsman Cancer Institute, University of Utah, Salt Lake City, Utah, United States of America; 65 Fox Chase Cancer Center, Philadelphia, Pennsylvania, United States of America; 66 Centre for Genetic Epidemiology, University of Melbourne, Melbourne, Australia; 67 Department of Epidemiology, Mailman School of Public Health, Columbia University, New York, New York, United States of America; 68 Division of Hematology-Oncology, Beth Israel Deaconess Medical Center, Boston, Massachusetts, United States of America; 69 Department of Clinical Biochemistry, Rigshospitalet, Copenhagen University Hospitalet, Copenhagen, Denmark; 70 Clinical Genetics Branch, National Cancer Institute, Rockville, Maryland, United States of America; 71 Human Genetics Group, Human Cancer Genetics Programme, Spanish National Cancer Research Centre, Madrid, Spain; 72 Institute of Biology and Molecular Genetics, Universidad de Valladolid (IBGM-UVA), Valladolid, Spain; 73 Oncology Service, Hospital Clínico Universitario Lozano Blesa, Zaragoza, Spain; 74 Human Genetics Group and Genotyping Unit, Human Cancer Genetics Programme, Spanish National Cancer Research Centre, Madrid, Spain; 75 City of Hope Cancer Center, Duarte, California, United States of America; 76 Dana Farber Cancer Institute, Harvard University, Boston, Massachusetts, United States of America; 77 Molecular Genetics of Breast Cancer, Deutsches Krebsforschungszentrum (DKFZ), Heidelberg, Germany; 78 Genetic Medicine, Manchester Academic Health Sciences Centre, Central Manchester University Hospitals NHS Foundation Trust, Manchester, United Kingdom; 79 Oncogenetics Team, The Institute of Cancer Research and Royal Marsden NHS Foundation Trust, London, United Kingdom; 80 Clinical Genetics, Guy's and St. Thomas' NHS Foundation Trust, London, United Kingdom; 81 Oxford Regional Genetics Service, Churchill Hospital, Oxford, United Kingdom; 82 Nottingham Clinical Genetics Service, Nottingham University Hospitals NHS Trust, Nottingham, United Kingdom; 83 Leicestershire Clinical Genetics Service, University Hospitals of Leicester NHS Trust, Leicester, United Kingdom; 84 Women's Cancer Program, Department of Medical Oncology, Fox Chase Cancer Center, Philadelphia, Pennsylvania, United States of America; 85 Centre of Familial Breast and Ovarian Cancer, Department of Gynaecology and Obstetrics and Centre for Integrated Oncology (CIO), University Hospital of Cologne, Cologne, Germany; 86 Department of Gynaecology and Obstetrics, Division of Tumor Genetics, Klinikum rechts der Isar, Technical University Munich, Munich, Germany; 87 Department of Gynaecology and Obstetrics, University Hospital of Schleswig-Holstein, Christian-Albrechts University Kiel, Kiel, Germany; 88 Institute of Cell and Molecular Pathology, Hannover Medical School, Hannover, Germany; 89 Program in Cancer Biology and Genetics, Memorial Sloan-Kettering Cancer Center, New York, New York, United States of America; Stanford University School of Medicine, United States of America

## Abstract

The considerable uncertainty regarding cancer risks associated with inherited mutations of *BRCA2* is due to unknown factors. To investigate whether common genetic variants modify penetrance for *BRCA2* mutation carriers, we undertook a two-staged genome-wide association study in *BRCA2* mutation carriers. In stage 1 using the Affymetrix 6.0 platform, 592,163 filtered SNPs genotyped were available on 899 young (<40 years) affected and 804 unaffected carriers of European ancestry. Associations were evaluated using a survival-based score test adjusted for familial correlations and stratified by country of the study and BRCA2*6174delT mutation status. The genomic inflation factor (λ) was 1.011. The stage 1 association analysis revealed multiple variants associated with breast cancer risk: 3 SNPs had p-values<10^−5^ and 39 SNPs had p-values<10^−4^. These variants included several previously associated with sporadic breast cancer risk and two novel loci on chromosome 20 (rs311499) and chromosome 10 (rs16917302). The chromosome 10 locus was in *ZNF365*, which contains another variant that has recently been associated with breast cancer in an independent study of unselected cases. In stage 2, the top 85 loci from stage 1 were genotyped in 1,264 cases and 1,222 controls. Hazard ratios (HR) and 95% confidence intervals (CI) for stage 1 and 2 were combined and estimated using a retrospective likelihood approach, stratified by country of residence and the most common mutation, BRCA2*6174delT. The combined per allele HR of the minor allele for the novel loci rs16917302 was 0.75 (95% CI 0.66–0.86, 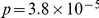) and for rs311499 was 0.72 (95% CI 0.61–0.85, 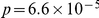). *FGFR2* rs2981575 had the strongest association with breast cancer risk (per allele HR = 1.28, 95% CI 1.18–1.39, 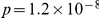). These results indicate that SNPs that modify *BRCA2* penetrance identified by an agnostic approach thus far are limited to variants that also modify risk of sporadic *BRCA2* wild-type breast cancer.

## Author Summary

The risk of breast cancer associated with *BRCA2* mutations varies widely. To determine whether common genetic variants modify the penetrance of *BRCA2* mutations, we conducted the first genome-wide association study of breast cancer among women with *BRCA2* mutations using a two-stage approach. The major finding of the study is that only those loci known to be associated with breast cancer risk in the general population, including *FGFR2* (rs2981575), modified *BRCA2*-associated risk in our high-risk population. Two novel loci, on chromosomes 10 in *ZNF365* (rs16917302) and chromosome 20 (rs311499), were shown to modify risk in *BRCA2* mutation carriers, although not at a genome-wide level of significance. However, the *ZNF365* locus has recently independently been associated with breast cancer risk in sporadic tumors, highlighting the potential significance of this zinc finger-containing gene in breast cancer pathogenesis. Our results indicate that it is unlikely that other common variants have a strong modifying effect on *BRCA2* penetrance.

## Introduction

After more than a decade of clinical testing for mutations of *BRCA1* and *BRCA2*, there remains considerable uncertainty regarding cancer risks associated with inherited mutations of these genes. This variable penetrance is most striking for *BRCA2* [1]–[4], and it affects medical management [5]. Women with the same *BRCA2* mutation may develop breast, ovarian or other cancers at different ages or not at all [6]. In a segregation analysis of families identified through breast cancer cases diagnosed before age 55, the residual familial clustering after accounting for *BRCA1* and *BRCA2* mutations could be explained by a large number of low penetrance genes with multiplicative effects on breast cancer risk [7], [8]. A candidate gene approach in *BRCA2* mutation carriers led to the discovery of loci that modify the penetrance of *BRCA2* mutations, such as *RAD51* 135 G>C [9] and perhaps *CASP8* [10], [11] and *IGFBP2* [12], if replicated. To investigate whether other common single nucleotide polymorphisms (SNP), copy number variants (CNV), or copy number polymorphisms (CNP) modify penetrance for *BRCA2* mutation carriers, we undertook a two-staged genome-wide association study (GWAS) in *BRCA2* mutation carriers from the international Consortium for Investigators of Modifiers of *BRCA1/2* (CIMBA) and other international studies. We hypothesized that an agnostic search for breast cancer loci in an enriched population of *BRCA2* mutation carriers, the first among this high risk population, would provide greater power than a sporadic population of equal number, and would yield associations specific to *BRCA2* carriers and/or the general population.

## Results

### Stage 1 and Stage 2 Genotyping

In stage 1, genotype data were available for 899 young (<40 years) affected and 804 older (>40 years) unaffected carriers of European ancestry after quality control filtering and removal of ethnic outliers (Figure S1). A total of 592,163 filtered SNPs genotyped using the Affymetrix Genome-Wide Human SNP Array 6.0 platform passed quality control assessment. In stage 1, comparison of the observed and expected distributions (quantile-quantile plot: Figure S2) showed little evidence for an inflation of the test statistics (genomic inflation factor λ = 1.01), thereby excluding the possibility of significant hidden population substructure, cryptic relatedness among subjects or differential genotype calling between *BRCA2* affected and *BRCA2* unaffected carriers. Multiple variants were found to be associated with breast cancer risk (Figure S3): 3 SNPs had p<10^−5^ and 39 SNPs had p<10^−4^. The most significant association (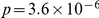) was observed for *FGFR2* rs2981582 (Table 1), a variant previously shown to be associated with increased risk of *BRCA2*-related breast cancer [13]. A positive association was also observed with rs3803662 (Table 1), near *TOX3*, which has also been associated with sporadic breast cancer risk [13].

**Table 1:** Estimates of breast cancer association for loci (two confirmatory loci at *FGFR2* and *TOX3*, and two novel loci with stage 1 and 2 combined of p<10^−4^) among *BRCA2* mutation carriers in a two-staged genome-wide association study.

Gene	SNP	Chr.	Stage 1			Stage 2			Stage 1 and 2 Combined			
			N (Controls/Cases)	p-value^2^	HR (95% CI)^1^	N (Controls/Cases)	HR (95% CI)^1^	p-value^2^	N (Controls/Cases)	MAF	HR (95% CI)^1^	p-value^2^
*FGFR2*	rs2981575	10	794/892	6.0×10^−6^	1.30 (1.16–1.45)	1,222/1,263	1.26 (1.11–1.43)	4.4×10^−4^	2,016/2,155	0.42	1.28 (1.18–1.39)	1.2×10^−8^
*TOX3*	rs3803662	16	804/899	5.8×10^−3^	1.19 (1.05–1.34)	1,222/1,263	1.22 (1.07–1.39)	2.8×10^−3^	2,026/2,162	0.29	1.20 (1.10–1.31)	4.9×10^−5^
*ZNF365*	rs16917302	10	804/898	1.8×10^−5^	0.67 (0.56–0.80)	1,222/1,264	0.85 (0.70–1.04)	0.14	2,026/2,162	0.11	0.75 (0.66–0.86)	3.8×10^−5^
*GMEB2*, *Etc.* 3	rs311499	20	792/882	3.5×10^−5^	0.60 (0.47–0.78)	1,209/1,255	0.84 (0.67–1.06)	0.13	2,001/2,137	0.07	0.72 (0.61–0.85)	6.6×10^−5^

1 p-value was calculated based on the 1-degree of freedom score test statistic stratified by country of study and 6174delT (c.5946delT) mutation status, and modified to allow for the non-independence among related individuals.

2 Per allele hazard ratios (HR) (i.e., multiplicative model) were estimated on the log scale, assuming independence of age, using the retrospective likelihood. All analyses were stratified by country of residence and 6174delT (c.5946delT) mutation status, and used calendar-year- and cohort-specific breast cancer incidence rates for *BRCA2*. The combined stage 1 and stage 2 analyses were also stratified by stage.

3 The region also includes other possible genes including *SRMS*, *PTK6*, *STMN3*, and *TNFRSF6* among others.

Using the stage 1 data, we also performed a GSEA as implemented in MAGENTA [14] to evaluate whether a functionally-related set of genes relevant to *BRCA2* function (Table S1) was enriched for relative risk associations (see Statistical Methods). The 59 genes selected are related to the Fanconi anemia pathway [15] as well as other pathways reported in the literature to regulate or interact with *BRCA1/2* [16]. These showed no enrichment of associations with the breast cancer risk (p = 0.56). In addition, eight of 125 known cancer susceptibility alleles identified by previous GWAS of other cancers [17] were associated with *BRCA2* modification in the current study, a number not greater than expected (Kolmogorv-Smirnov p = 0.60) by chance alone. Of the 113 most significantly associated SNPs (p<10^−3^) in our study, three showed significant association (p<0.05) with *BRCA1*-associated breast cancer risk in a complimentary GWAS [18].

**Figure 1:**
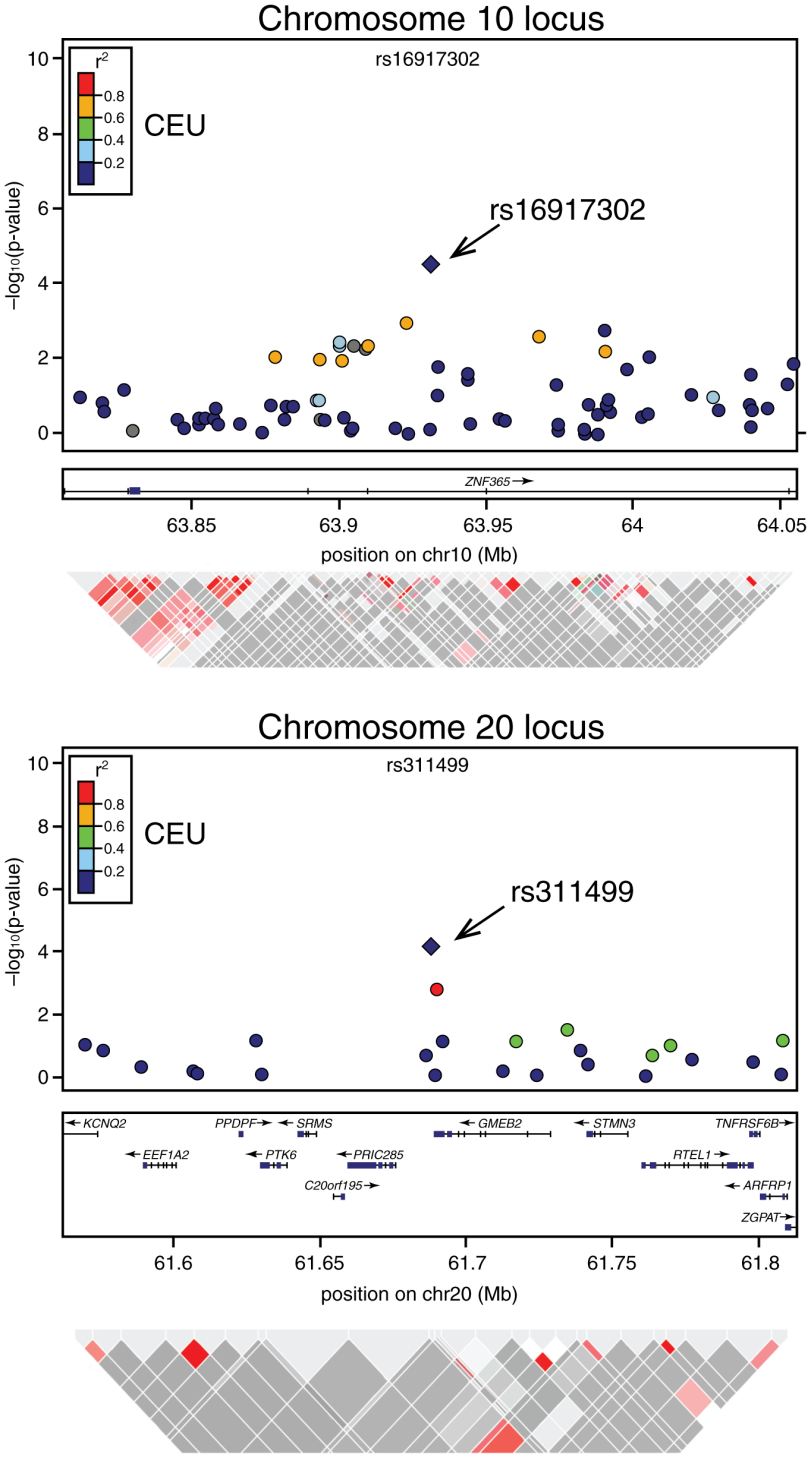
Association signals, genetic structure, and linkage disequilibrium of the novel modifier loci of *BRCA2* penetrance in the regions surrounding rs1691730 on chromosome 10 and rs311499 on chromosome 20. The color of the dots indicates linkage disequilibrium (LD; based on r2 values) in the CEU population (as per scale). Triangle plots below represent LD from actual data of *BRCA2* carries in the study.

In the combined stage 1 and stage 2 results, four independent SNPs (pairwise 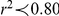) were associated with increased risk of breast cancer risk with p-values<10^−4^ (Table 1). Previously identified breast cancer susceptibility loci [13], [19], [20] had the most significant associations among *BRCA2* mutation carriers (*FGFR2*: per allele 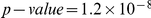 and *TOX3*: per allele 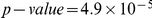). Novel loci, rs16917302 on chromosome 10 and rs311499 on chromosome 20, had HRs in stage 2 that were in the same direction as those observed for stage 1 (Figure 1, Table 1), but were smaller in magnitude (HR = 0.67 (95% CI:0.56–0.80) vs. 0.85 (95% CI: 0.70–1.04) for rs16917302; HR = 0.60 (95%CI:0.50–0.78) vs. 0.84 (95%CI: 0.67–1.06) for rs311499) perhaps reflecting a “winner's curse” effect” [21]. The associations for these SNPs were not statistically significant in stage 2 (Table 1). In the combined stage 1 and stage 2 dataset, the C allele of rs16917302 was associated with lower risk of breast cancer (per allele HR = 0.75, 95% CI 0.66–0.86; 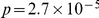; Table 1), and the C allele of rs311499 was associated with a reduced risk (per allele HR = 0.72, 95% CI 0.61–0.85; 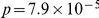; Table 1). A full list of stage 2 results can be found in Table S2. Using the combined stage 1 and stage 2 data, there was no evidence that the HR for SNP rs16917302 changes with age (p = 0.63), but there was some evidence that the per-allele HR for rs311499 may increase with age (p = 0.034).

### Copy Number Variant Analysis

We also examined the association of both high-frequency CNPs and low-frequency CNVs to case-control status using the stage 1 data. After performing standard quality control measures including a minor allele frequency (MAF) threshold of 5%, we identified 191 polymorphisms with reliable genotypes. No associations were found between CNVs and the phenotype; there was no inflation or deflation of the test statistic, and the best p-value was 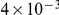. We similarly assessed less common CNPs, and found neither the overall burden of events (or any subclass thereof, such as large deletions overlapping genes) nor any specific locus associated with breast cancer risk (Figure S4).

### Excess Sharing in Genetic Isolates and Outbred Populations Analyzed

Because of the prior evidence of significant LD extent around the 6174delT (c.5946delT) founder mutation in the Ashkenazi Jewish population [22], we explored the potential excess sharing of the genome compared to the *BRCA2* region in both Ashkenazi Jewish and non-Jewish European ancestries. Using GERMLINE [23], shared segments of greater than 5 cM were computed based on the imputed genotype dataset. In the *BRCA2* region, we observed a significant excess of sharing amongst both Ashkenazi (n = 304) and non-Jewish (n = 1331) individuals compared to samples from an autism study (n = 808) suggesting common founders for *BRCA2* mutations. Examining sites across the genome every 2.5 cM (excluding telomere and centromere regions), we observed possible pairs share segments greater than 5 cM that on average 0.005% (u = 50.17, s.d = 55.5, max = 491) for non-Jewish individuals and 0.12% (u = 141.11, s.d = 57.32, max = 525) for Ashkenazi Jewish individuals. Comparing cases and controls, we did not observe a significant difference in number of pairs of samples sharing segments greater than 5cM across the genome excluding chromosome 13. That is, there was no evidence of overall excess sharing across the genome other than for the *BRCA2* locus within the Ashkenazi Jewish and non-Ashkenazi Jewish populations in the study.

## Discussion

In this GWAS of *BRCA2* mutation carriers, the first in this high risk population, we found previously identified breast cancer susceptibility loci modified risk of *BRCA2*-associated breast cancer with similar magnitude of association. Although *FGFR2* (rs2981575) was the only locus to reach genome-wide statistical significance, novel loci, rs16917302 and rs10509168 were each associated with breast cancer risk.

rs16917302 is located on chromosome 10, in the zinc finger protein 365 gene (*ZNF365*). A recent multistage GWAS of 15,992 sporadic breast cancer cases and 16,891 controls also observed an inverse association (per allele OR = 0.82, 95% CI 0.82–0.91, 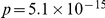) between breast cancer risk and rs10509168, a SNP 18kb from rs16917302 (pairwise 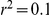) and located in intron 4 of *ZNF365* [*24*]. Of the 3,659 cases and 4,897 controls in phase 1 of that study, imputation revealed that the locus identified in our *BRCA2* study, rs16917302, was significantly associated with risk for breast cancer (p = 0.02) (Easton DF, personal communication). The second novel SNP in the current study, rs311499, is located on chromosome 20, within a region containing several possible candidate genes including *GMEB2*, *SRMS*, *PTK6*, *STMN3*, and *TNFRSF6*. The functional significance of both of these regions with breast carcinogenesis is unknown; further research is warranted.

There was some evidence that the HR associated with rs311499 may change with age. We also observed that the stage 1 HR for this SNPs was larger in magnitude compared to the stage 2 HR, consistent with a winner's curse effect [21]. Since stage 1 of our experiment included mostly *BRCA2* mutation carriers diagnosed at a young age, and stage 2 mutation carriers diagnosed an older age, the “winner's curse” and age-specific effects are confounded and may be difficult to distinguish. Fitting the age-dependent HR model for SNP rs311499 using the stage 2 data yielded no significant variation in the HR by age (p = 0.47), but the sample size for this analysis was relatively small. Future larger studies should aim to clarify this.

Mutations in known genes (*BRCA1*, *BRCA2*, *TP53*, *CHEK2*, *PTEN*, and *ATM*) explain only 20–25% of the familial clustering of breast cancer; the residual familial clustering may be explained by the existence of multiple common, low-penetrance alleles (‘polygenes’) [25]. Perhaps because the majority of *BRCA2*-associated breast tumors are estrogen receptor (ER)-positive, as are the majority of non-hereditary breast cancers [26], risk alleles for sporadic breast cancer are more likely to be modifiers of risk of *BRCA2*-associated hereditary breast cancer. Of the seven GWAS-identified breast cancer-associated SNPs examined in a *BRCA2* background [13], [19], [20], SNPS in *FGFR2* (rs2981575), *TOX3* (rs3803662), *MAP3K1* (rs889312), and *LSP1* (rs3817198) have been shown to modify *BRCA2* penetrance, in contrast with *BRCA1* tumors, in which only two of these same SNPs (based on a 2 degrees of freedom model) modified risk of these largely ER-negative tumors [26]. As previously noted [13], [20], the stage 1 HRs among *BRCA2* mutation carriers, reported here, were nearly identical to odds ratio estimates observed in sporadic breast cancer studies, consistent with a simple multiplicative interaction between the *BRCA2* mutant alleles and the common susceptibility SNPs. If replicated, the two additional SNPs identified here would only explain about 1.7% of the variance in breast cancer risk among *BRCA2* mutation carriers. Taken together, the combined effects of all the common and putative risk modifiers in this study only account for ∼4% of the variance of *BRCA2* mutations, compared with 1.1% for the single *RAD51* 135 G>C variant, which is rare and biologically-linked to *BRCA2* function, as shown by candidate gene studies [9]. Thus, the common alleles that modify risk in *BRCA1* and *BRCA2* backgrounds appear to have comparable associated risks in sporadic ER-positive and ER-negative tumors, respectively [18]. While individual SNPs are unlikely to be used to guide radiographic screening and risk-reducing surgical strategies, the combined effect of these SNPs may ultimately be used for the tailor management of subsets of *BRCA* mutation carriers [5].

While we took great efforts to collect all of the possible known *BRCA2* mutation carriers, there were insufficient numbers to stratify by race and *BRCA2* mutations with the exception of BRCA2*6174delT mutations. Due to the small numbers of women of non-European ancestry who have participated in the individual studies represented here, the current analysis was based only on women who had genetic backgrounds consistent with HapMap CEU samples. While we expect that SNPs identified among women of European ancestry might also be applicable to women of other genetic backgrounds, additional research in these populations will be needed. Similarly, the observed associations represented across all types of mutations, and specifically a weighted average of BRCA2*6174delT and non-delT mutations. It is possible that the observed associations may only modify the penetrance of specific *BRCA2* mutations due to differential effects on function or differences in genetic background. Our analysis was stratified on the basis of the most common *BRCA2* mutation, BRCA2*6174delT, which is prevalent in individuals with an Ashkenazi Jewish ancestry. Large numbers of mutation carriers will be necessary to calculate mutation-specific estimates. In addition, there was a drop-out of SNPs in the two phases of this study. While we were able to achieve a representative coverage of the genome, it is also possible that additional studies using denser arrays may provide further information.

As expected, we observed associations with some of the major common genetic variants seen in genome-wide scans of breast cancer in a non-*BRCA1/2* mutation background. However, we found no evidence for loci with stronger effects than *FGFR2*. Although we observed an association with a novel locus at *ZNF365* that appears also to be a risk factor for sporadic breast cancer, overall, our results suggest that there are no common variants with major effects (i.e., OR>2.0) that are specific in *BRCA2* carriers. Similarly, in a recent report of SNPs from sporadic breast cancer GWAS genotyped in a restricted set of *BRCA1/2* carriers [27], loci in *LOC134997* (rs9393597: per allele HR = 1.55, 95% CI 1.25–1.92, 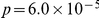) and *FBXL7* (rs12652447: HR = 1.37, 95% CI 1.16–1.62, 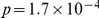) were associated with *BRCA2* breast cancer risk with p-values weaker than *FGFR2* reported here (per allele 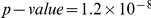), although the magnitudes of the associations were slightly stronger than *FGFR2* (HR = 1.28). Although these SNPs were not in our genotyped panel of SNPs at stage 1, imputation results indicate that SNP rs9393597 has a p-value of 0.008 and SNP rs12652447 a p-value of 0.04 for association with breast cancer risk for the *BRCA2* mutation carriers in our stage1. However, there is substantial overlap between our study and the study of Wang et al. [27].

Replication in larger datasets will be necessary to precisely estimate the magnitude of the associations of suspected loci identified from our study, candidate gene analysis [10]–[12], and other selection approaches [27]. It is of interest, however, that when utilizing an agnostic approach in *BRCA2* mutation carriers in this study, the major determinants of risk variation in mutation carriers are those that also modify risk in subsets of sporadic, *BRCA1/2* wild type, breast cancer. However, it remains possible that unique variants with smaller effects, or rarer variants (not evaluated in this experiment), may be specific modifiers of breast cancer risk in *BRCA2* carriers. Their detection would require study populations much larger than the current analysis, which is presently the largest such cohort assembled.

## Materials and Methods

### Study Subjects

#### Ethics statement

All carriers were recruited to studies (Table 2) at the host institutions under IRB-approved protocols.

**Table 2:** Description of affected and unaffected carriers selected for *BRCA2* GWAS Stage 1 and 2.

	Stage 1				Stage 2			
	Affected (n = 1,156)		Unaffected (n = 1,038)		Affected (n = 1,524)		Unaffected (n = 1,508)	
Factor	N	%	N	%	N	%	N	%
**Age at Censoring**								
<40	763	66.7	11	1.1	368	23.7	1007	66.0
40–44	308	26.9	230	22.2	225	14.5	119	7.8
45–49	72	6.3	232	22.4	334	21.5	131	8.6
50–54	1	0.1	176	17.0	286	18.4	90	5.9
55–59	0	0.0	138	13.3	164	10.5	73	4.8
60+	0	0.0	248	24.0	178	11.4	105	6.9
**Self-reported Ethnicity**								
Unknown	125	10.9	80	7.7	329	21.2	293	19.2
Caucasian	873	76.3	723	69.9	1037	66.7	1036	67.9
Ashkenazi Jewish	146	12.8	232	22.4	189	12.2	196	12.9
**DelT Mutation**								
Carrier	161	14.1	271	26.2	233	15.0	239	15.7
Non-carrier	983	85.9	764	73.8	1322	85.0	1286	84.3
**Country of Study**								
Australia	109	9.5	82	7.9	149	9.6	180	11.8
Canada	98	8.6	172	16.6	55	3.5	82	5.4
Denmark	0	0.0	0	0.0	43	2.8	32	2.1
France	52	4.5	25	2.4	172	11.1	50	3.3
Finland	27	2.4	27	2.6	32	2.1	27	1.8
Germany	68	5.9	31	3.0	116	7.5	54	3.5
Iceland	25	2.2	9	0.9	81	5.2	6	0.4
Israel	49	4.3	87	8.4	77	5.0	86	5.6
Italy	110	9.6	44	4.3	98	6.3	62	4.1
Spain	107	9.4	71	6.9	99	6.4	136	8.9
Sweden	13	1.1	13	1.3	11	0.7	15	1.0
The Netherlands	15	1.3	26	2.5	117	7.5	201	13.2
United Kingdom	181	15.8	179	17.3	125	8.0	168	11.0
USA	290	25.4	290	28.0	380	24.3	426	27.9
**Study**								
BCFR-Australia	19	1.7	5	0.5	12	0.8	10	0.7
BCFR-NCCC	12	1.0	1	0.1	5	0.3	2	0.1
BCFR-Ontario	29	2.5	28	2.7	16	1.0	17	1.1
BCFR-UT	18	1.6	18	1.7	11	0.7	47	3.1
BCFR-FCCC	2	0.2	1	0.1	14	0.9	10	0.7
BCFR-NY	4	0.3	5	0.5	26	1.7	16	1.0
BIDMC	10	0.9	20	1.9	7	0.5	12	0.8
CBCS	0	0.0	0	0.0	43	2.8	32	2.1
CGB_NCI	7	0.6	15	1.4	14	0.9	43	2.8
CNIO	49	4.3	33	3.2	40	2.5	56	3.7
COH	30	2.6	13	1.3	21	1.4	16	1.0
DFCI	14	1.2	22	2.1	10	0.6	24	1.6
DKFZ	7	0.6	5	0.5	8	0.5	7	0.5
EMBRACE	178	15.6	173	16.7	123	7.9	161	10.6
FCCC	14	1.2	10	1.0	12	0.8	9	0.6
GC-HBOC	61	5.3	26	2.5	108	6.9	47	3.1
GEMO	52	4.5	25	2.4	172	11.0	50	3.3
GOG	64	5.6	51	4.9	57	3.7	91	6.0
HCSC	27	2.4	20	1.9	34	2.2	35	2.3
HEBON	10	0.9	17	1.6	103	6.6	172	11.3
HEBCS	27	2.4	27	2.6	32	2.1	27	1.8
ICO	31	2.7	18	1.7	25	1.6	45	3.0
ILUH	26	2.3	9	0.9	81	5.2	6	0.4
IOVHBOCS	19	1.7	7	0.7	44	2.8	20	1.3
kConFab	88	7.6	77	7.3	137	8.7	168	11.0
LUMC	5	0.4	9	0.9	14	0.9	29	1.9
MAGIC-UC	2	0.2	2	0.2	0	0.0	3	0.2
MAGIC-UCI	6	0.5	9	0.9	21	1.4	22	1.4
MAYO	5	0.4	14	1.4	51	3.3	24	1.6
MBCSG	91	8.0	37	3.6	54	3.5	42	2.8
MSKCC	51	4.5	61	5.9	52	3.3	47	3.1
NICC	28	2.4	60	5.8	46	3.0	67	4.4
OCGN	62	5.4	60	5.8	35	2.2	36	2.4
OSU CCG	11	1.0	8	0.8	9	0.6	8	0.5
SMC	21	1.8	27	2.6	31	2.0	19	1.2
SWE-BRCA	13	1.1	13	1.3	11	0.7	15	1.0
UCSF	10	0.9	6	0.6	12	0.8	8	0.5
UKGRFOCR	2	0.2	6	0.6	2	0.1	7	0.5
UPENN	33	2.9	13	1.3	58	3.7	46	3.0
WCRI	6	0.5	84	8.1	4	0.3	29	1.9

#### Selection of affected individuals and controls

A total of 6,272 *BRCA2* carriers from 39 studies (Table 2) and 14 countries contributed DNA samples for this project. With the exception of NICC, all studies are members of the Consortium of Investigators of Modifiers of BRCA1/2 (CIMBA) [28]. Recruitment of carriers were conducted predominantly through cancer genetics clinics, and enrolled through national or regional efforts. Other studies were recruited through population-based or community-based ascertainments. All subjects provided written informed consent. Eligible female carriers were aged 18 years or older, were self-reported ‘white’, and had mutations in *BRCA2*. Data were available on age at study recruitment, age at cancer diagnosis, age of bilateral prophylactic mastectomy, *BRCA1/2* mutation description, and self-reported ethnicity. Only a limited number of cases had detailed information on tumor characteristics (e.g., estrogen and progesterone receptor status); therefore, subtype analyses were not performed at this stage.

### Genotyping and Quality Control

#### Stage 1 Affymetrix genotyping

All eligible DNA samples provided by participating centers were subjected to a rigorous quality control assessment, including measures of overall DNA quality and quantity. A total of 1,156 young (≤50 years) affected women and 1,038 unaffected women with high quality DNA samples were selected (Table 2). For time efficiency, stage 1 genotyping occurred in two phases: phase 1 included 421 cases and 404 controls and phase 2 included 735 cases and 634 controls.

Prior to the genome-wide scan, we genotyped five SNPs previously genotyped by the CIMBA study centers as a pre-filter for sample identification. Thirty-one samples (Figure S1) were discordant in the two genotyping rounds and were excluded from further analysis.

The genotyping for the stage 1 GWAS was performed on 2,163 eligible carriers using the Affymetrix 6.0 GeneChip array that included 906,622 SNPs (Figure S1). To further monitor the identity of the DNA samples, a fingerprinting panel of 14 SNPs with a minor allele frequency >10% in HapMap European individuals were genotyped on all samples, using Sequenom iPLEX, before and after Affymetrix genotyping. The AMG gender assay was used for gender assessment. As an additional quality control measure, cases and controls were interleafed on each plate to eliminate technical bias. Each plate also included one HapMap CEU DNA sample.

The DNA samples and genotyping calls for both phases of stage 1 were filtered through a series of data quality control parameters using the Birdseed module of the Birdsuite software developed at Broad Institute [29]. Among the 2,163 samples genotyped in the stage 1 GWAS, 253 failed to hybridize to the chip due to poor DNA quality and were excluded (Figure S1). Fifty-five samples were dropped with call rates <95%. Three samples were contaminated, 43 were identified by genotyping to be duplicates, and 4 were male; all were dropped from analyses.

SNPs were also filtered using Birdseed and were removed if monomorphic or >10% missing (n = 38,962), genotype call rates <95% (n = 50,810), minor allele frequencies <1% (n = 104,792), departures from Hardy-Weinberg Equilibrium (p<10^−6^; n = 1,090), differential missingness with respect to phenotype (p<10^−3^; n = 275), and differential missingness with respect to nearby SNPs (p<10^−10^; n = 22,065). A total of 6,212 SNPs had different missingness patterns in phase 1 compared to phase 2, and were excluded. Since we found that significant missingness correlated to SNPs mapping to longer fragments of Affymetrix 6.0 digestion products, we also removed the SNPs on fragments longer than 1000bp (n = 85,990).

With the remaining 1,805 carriers and 596,426 SNPs, an iterative process proceeded to drop all individuals with low call rates (<95%), high autosomal heterozygosity rates (false discovery rate <0.1%), and high identity by descent scores (≥0.95) and to drop all SNPs with minor allele frequencies <1% and SNP call rates <95% until the final run contained individuals above the individual and SNP filter thresholds (n = 1,747 samples and 592,566 SNPs). A more stringent HWE filter (p<10^−7^) was then applied and 403 additional SNPs were dropped. Nine individuals with missing mutation descriptions were removed.

Finally, principal components analysis was used to identify the ethnic outliers (Figure S5). A total of 1,743 *BRCA2* mutation carriers and the HapMap3 data for 210 individuals of European (CEU), Han Chinese (CHB), and Yoruba (YRI) African descent were available for multidimensional scaling using the genomic kinship matrix estimated using a set of 53,641 autosomal and uncorrelated SNPs. A cut-off of >11% was used to exclude samples with non-CEU ancestry (n = 35). Genotype-phenotype association analyses were based on 1,703 (899 young affected and 804 unaffected) *BRCA2* mutation carriers and 592,163 SNPs, covering 85% of the common HapMap 3 SNPs (imputed with 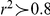 (see below), including 64% of the markers that were removed in the QC process).

Where directly genotyped data were not available, probabilities were imputed with Beagle.3.0.2 (using the default parameters) using CEU+TSI samples on HapMap3 release2 B36 as the reference panel (410 chromosomes, 1.4 M SNPs).

#### Stage 2 Sequenom iPLEX genotyping

The primary SNP selection strategy was based on the results of the kinship-adjusted score test of 592,163 GWAS genotyped SNPs. From stage 1, a total of 79 top independent regions (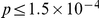) with pairwise r^2^ values<0.80 were selected for genotyping in stage 2 (Figure S6). For the top 10 SNPs if available, an additional correlated SNP (pairwise 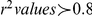; n = 5) was selected to serve as genotyping backup. The remaining SNPs for stage 2 were selected based on two alternate strategies. First, we added the 14 (as well as *FGFR2* counted in the top 10 SNPs above) confirmed breast cancer SNPs from prior independent GWAS of sporadic breast cancer. Second, we also selected the 15 top independent regions (pairwise 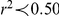) based on the ranking of the p-values from a logistic regression analysis of 1.5 million imputed SNPs. In total for stage 2 replication phase, we selected 113 SNPs and 1,524 breast cancer carriers and 1,508 control carriers (Table 2) for genotyping using the Sequenom iPLEX platform.

Samples were excluded for call rates ≤95% (n = 476), duplication in stage 2 (identity by state (IBS)∼1.0; n = 43), duplication in stage 1 and 2 (IBS; n = 25), lack of complete phenotype data (n = 1), and insufficient country-specific numbers (n = 1; Figure S6). A total of 100 SNPs were successfully multiplexed into three pools; the remaining 13 SNPs were not genotyped. Genotyping QC filters excluded 15 SNPs due to call rates ≤90% (n = 14) and MAF<1% (n = 1). In summary, the final association analyses in stage 2 were based on 2,486 carriers (1,264 affected and 1,222 unaffected carriers) and 85 SNPs.

### Statistical Methods

#### Defining time at risk

Carriers were censored at the first breast or ovarian cancer or bilateral prophylactic mastectomy, whichever occurred first. Carriers who developed any cancer were censored at time of bilateral prophylactic mastectomy if it occurred more than a year prior to the cancer diagnosis (to avoid censoring at bilateral mastectomies related to diagnosis in which rounded ages were used). The remaining carriers were censored at the age of last observation. This was defined either by the age/date at interview or age at follow-up depending on the information provided by the participating center. Carriers censored at diagnosis of breast cancer were considered cases in the analysis. Mutation carriers censored at ovarian cancer diagnosis were considered unaffected. Carriers with a censoring/last follow-up age older than age 80 were censored at age 80 because there are no reliable cancer incidence rates for *BRCA1/2* carriers beyond age 80.

#### Genotype–phenotype associations

Analyses, based on 1,703 *BRCA2* mutation carriers and 592,163 SNPs, were performed within a survival analysis framework. Since the mutation carriers were not selected at random with respect to their disease status, standard methods of survival (e.g., Cox regression) may lead to biased estimates of relative risk [30]. Therefore, analyses were conducted by modeling the retrospective likelihood of the observed genotypes conditional on the disease phenotypes. The associations between genotype and breast cancer risk at both stages were assessed using the 1-degree of freedom score test statistic based on this retrospective likelihood, as previously described [9], [18]. All models were stratified by country of study and 6174delT (c.5946delT) mutation status, the most common *BRCA2* mutation in this study and a marker of the Ashkenazi Jewish population among Ashkenazi Jewish women [31]–[33]. Since the linkage disequilibrium structure among Ashkenazi Jewish people may differ from other mutation carriers [34], stratifying by the *6174delT provides additional control for population stratification. To allow for the non-independence among related individuals, an adjusted version of the score test was used in which the variance of the score was derived by taking into account the correlation between the genotypes [35], [36]. Analyses were performed in R using the GenABEL libraries [37] and custom written software.

To estimate the magnitude of the associations, the effect of each SNP was modelled either as a per allele hazard ratio (HR) (i.e., multiplicative model) or as separate HRs for heterozygotes and homozygotes, and these were estimated on the log scale. The HRs were assumed to be independent of age (i.e. we used a Cox proportional-hazards model). For the most significant novel associations this assumption was verified by adding a genotype-by-age interaction term to the model to fit models in which the HR changed with age. The retrospective likelihood was implemented in the pedigree-analysis software MENDEL [38] as previously described [9]. All analyses were stratified by country of residence and 6174delT (c.5946delT) mutation status, and used calendar-year- and cohort-specific breast cancer incidence rates for *BRCA2* [25]. The combined stage 1 and stage 2 analyses were also stratified by stage. Parameter estimates were obtained by maximising the retrospective likelihood. To allow for the non-independence among related mutation carriers, we used a robust variance estimation approach in order to obtain standard errors for the parameters [39], [40]. Related individuals were identified through a unique family identifier.

#### Copy number variant analysis

We also examined the association of both high-frequency and low-frequency copy number variants (CNV) to the age of diagnosis of breast cancer as a dichotomous trait using the stage 1 data [29]. We called known, common variants (copy number polymorphisms, CNPs) with Canary [29]. CNP alleles lower than 1% in frequency were removed, to maximize the number of the CNPs that were bi-allelic instead of multi-alleleic. CNPs were removed that had for call rate <95%, differential missingness by genotype (p<10^−3^), or departure from Hardy-Weinberg proportions (p<10^−3^). Post-QC, we had 191 high-quality genotyped polymorphisms. We used PLINK to assess association using logistic regression and the same ancestry covariates of no interest as with SNPs. We similarly assessed less common CNVs discovered by Birdseye [29] for association with age at diagnosis using PLINK [41]. Finally, we also looked specifically at CNVs overlapping the *BRCA2* gene itself using LOD scores and Birdseye.

#### Haplotype sharing analysis

We looked for evidence of excess sharing across the genome and the *BRCA2* region. Using GERMLINE [23], shared segments of greater than 5 cM were computed based on the imputed genotype dataset among both Ashkenazi (n = 304) and non-Jewish (n = 1,331) samples compared to samples from an autism study (n = 808) (Figure S3). Examining sites across the genome every 2.5 cM (excluding telomere and centromere regions), we computed the mean of the proportion, standard deviation, and the maximum values for non-Jewish and Ashkenazi women, respectively.

#### Gene Set Enrichment Analysis

We tested whether 59 genes known to regulate or interact with *BRCA2* [16] (Table S1) were enriched for associations with age of onset of breast cancer in *BRCA2* mutation carriers, using a new implementation of Gene Set Enrichment Analysis (GSEA) called Meta-Analysis Gene-Set Enrichment of variaNT Associations (MAGENTA) [14]. The 59 genes were compiled using a Pubmed abstract mining software, Chilibot [42], and were selected if they were related to the Fanconi anemia pathway [15] as well as others reported from literature to regulate or interact with *BRCA1/2* [43]. An association p-value was calculated for each gene in the genome, defined as the most-significant association p-value of all genotyped SNPs that lie within 110 kb upstream and 40 kb downstream to the gene's most extreme transcript boundaries, followed by correction for gene score confounders (gene size, number of SNPs per gene and linkage disequilibrium related properties). SNP association p-values were taken from the stage 1 GWAS. To compute a GSEA p-value for the *BRCA* gene set, the fraction of genes with an association p-value more significant than the 95 percentile of all gene p-values in the genome was compared to a null distribution, generated by randomly sampling gene-sets of identical size from the genome 10,000 times. Of the 59 *BRCA* interactors, two genes were assigned the same most significant SNP due to physical proximity in the genome. To prevent potential over-estimation of gene set enrichment due to physical clustering of genes in a gene set, we retained only one gene of each subset of genes assigned the same best SNP (the gene with the most significant gene p-value) for the analysis of both the real and permuted gene sets.

## Supporting Information

Figure S1 Data filtering of stage 1 *BRCA2* GWAS.
(0.57 MB TIF)

Figure S2 Quantile-quantile plot comparing expected distribution of chi-square values and observed chi-square values from a genome-wide scan of breast cancer cases and unaffected *BRCA2* carriers.
(0.31 MB TIF)

Figure S3 Manhattan plot of p-values by chromosomal position from a genome-wide scan of breast cancer cases and unaffected *BRCA2* carriers [Visualized using SVS7 (Goldenhelix)].
(0.51 MB TIF)

Figure S4 Quantile-quantile plot comparing expected distribution of p-values and observed p-values of association of common copy number polymorphisms (CNPs) from a genome-wide scan of breast cancer cases and unaffected *BRCA2* carriers.
(0.32 MB TIF)

Figure S5 Principal components analysis, including all eligible (after filtering) *BRCA2* stage 1 samples and HapMap samples.
(2.39 MB TIF)

Figure S6 Data filtering of stage 2 *BRCA2* GWAS.
(0.35 MB TIF)

Table S1 List of 59 *BRCA* interactors or regulators and their gene association p-values to breast cancer age of onset in *BRCA2* mutation carriers.
(0.13 MB DOC)

Table S2 Ranked results for the 85 SNPs successfully genotyped in stage 2, *BRCA2* GWAS.
(0.13 MB DOC)
