# Correction: Common Genetic Variants and Modification of Penetrance of *BRCA2*-Associated Breast Cancer

**DOI:** 10.1371/annotation/59ea8540-4e63-4f4a-a79e-f68765fdeac7

**Published:** 2010-11-17

**Authors:** Mia M. Gaudet, Tomas Kirchhoff, Todd Green, Joseph Vijai, Joshua M. Korn, Candace Guiducci, Ayellet V. Segrè, Kate McGee, Lesley McGuffog, Christiana Kartsonaki, Jonathan Morrison, Sue Healey, Olga M. Sinilnikova, Dominique Stoppa-Lyonnet, Sylvie Mazoyer, Marion Gauthier-Villars, Hagay Sobol, Michel Longy, Marc Frenay, GEMO Study Collaborators, Frans B. L. Hogervorst, Matti A. Rookus, J. Margriet Collée, Nicoline Hoogerbrugge, Kees E. P. van Roozendaal, HEBON Study Collaborators, Marion Piedmonte, Wendy Rubinstein, Stacy Nerenstone, Linda Van Le, Stephanie V. Blank, Trinidad Caldés, Miguel de la Hoya, Heli Nevanlinna, Kristiina Aittomäki, Conxi Lazaro, Ignacio Blanco, Adalgeir Arason, Oskar T. Johannsson, Rosa B. Barkardottir, Peter Devilee, Olofunmilayo I. Olopade, Susan L. Neuhausen, Xianshu Wang, Zachary S. Fredericksen, Paolo Peterlongo, Siranoush Manoukian, Monica Barile, Alessandra Viel, Paolo Radice, Catherine M. Phelan, Steven Narod, Gad Rennert, Flavio Lejbkowicz, Anath Flugelman, Irene L. Andrulis, Gord Glendon, Hilmi Ozcelik, OCGN, Amanda E. Toland, Marco Montagna, Emma D'Andrea, Eitan Friedman, Yael Laitman, Ake Borg, Mary Beattie, Susan J. Ramus, Susan M. Domchek, Katherine L. Nathanson, Tim Rebbeck, Amanda B. Spurdle, Xiaoqing Chen, Helene Holland, kConFab, Esther M. John, John L. Hopper, Saundra S. Buys, Mary B. Daly, Melissa C. Southey, Mary Beth Terry, Nadine Tung, Thomas V. Overeem Hansen, Finn C. Nielsen, Mark H. Greene, Phuong L. Mai, Ana Osorio, Mercedes Durán, Raquel Andres, Javier Benítez, Jeffrey N. Weitzel, Judy Garber, Ute Hamann, Susan Peock, Margaret Cook, Clare Oliver, Debra Frost, Radka Platte, D. Gareth Evans, Fiona Lalloo, Ros Eeles, Louise Izatt, Lisa Walker, Jacqueline Eason, Julian Barwell, Andrew K. Godwin, Rita K. Schmutzler, Barbara Wappenschmidt, Stefanie Engert, Norbert Arnold, Dorothea Gadzicki, Michael Dean, Bert Gold, Robert J. Klein, Fergus J. Couch, Georgia Chenevix-Trench, Douglas F. Easton, Mark J. Daly, Antonis C. Antoniou, David M. Altshuler, Kenneth Offit

The name of the 84th author contained an error. Mark I. Greene should be Mark H. Greene.

