# Supplementary figures and images for: Common Genetic Variants and Modification of Penetrance of BRCA2-Associated Breast Cancer
Source: PLoS Genet. 2010 Oct 28;6(10):e1001183. doi: 10.1371/journal.pgen.1001183 (PMC2965747; doi:10.1371/journal.pgen.1001183)

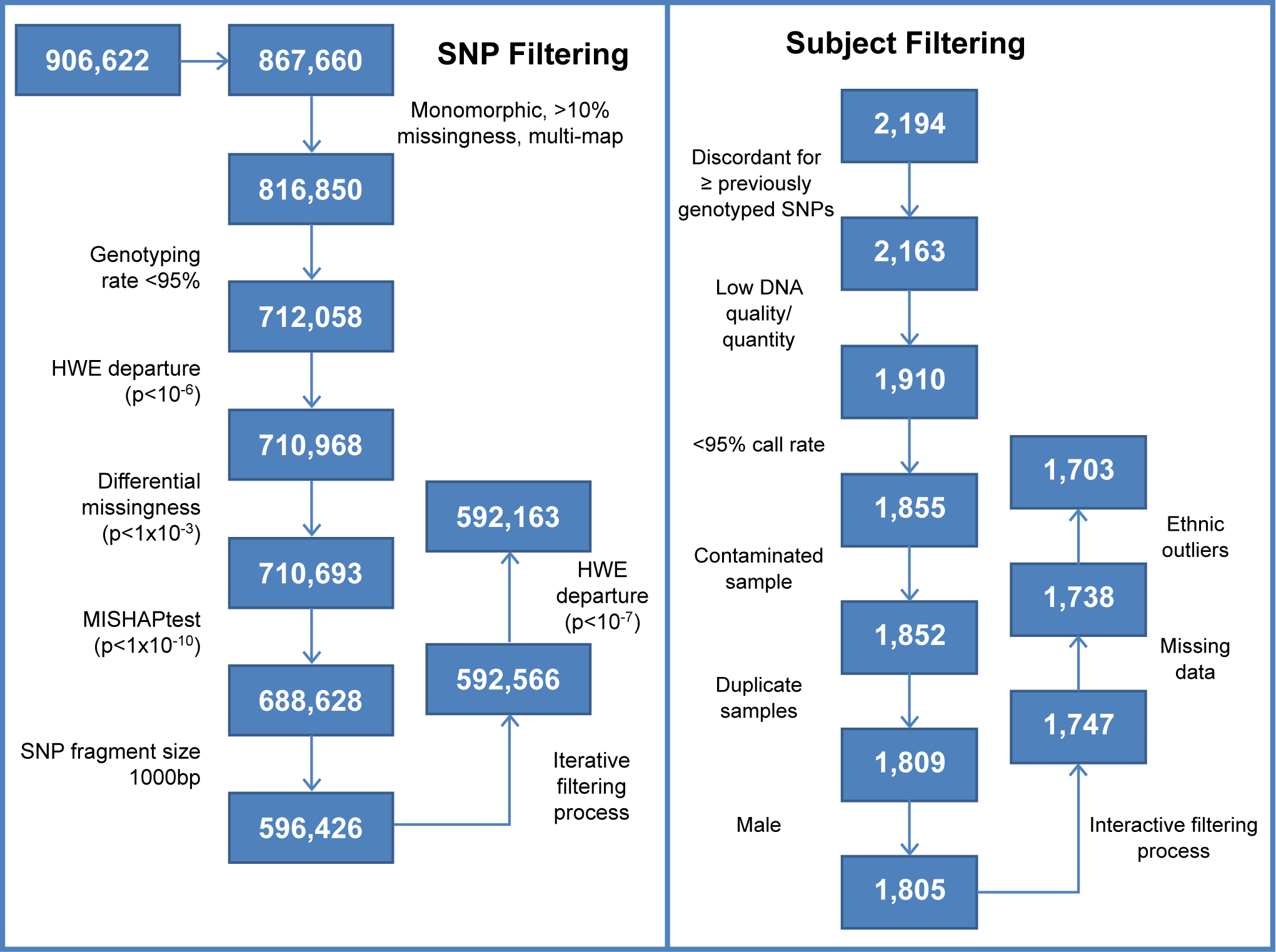

Supplement: Figure S1 — Data filtering of stage 1 BRCA2 GWAS. (0.57 MB TIF) [file pgen.1001183.s001.tif]

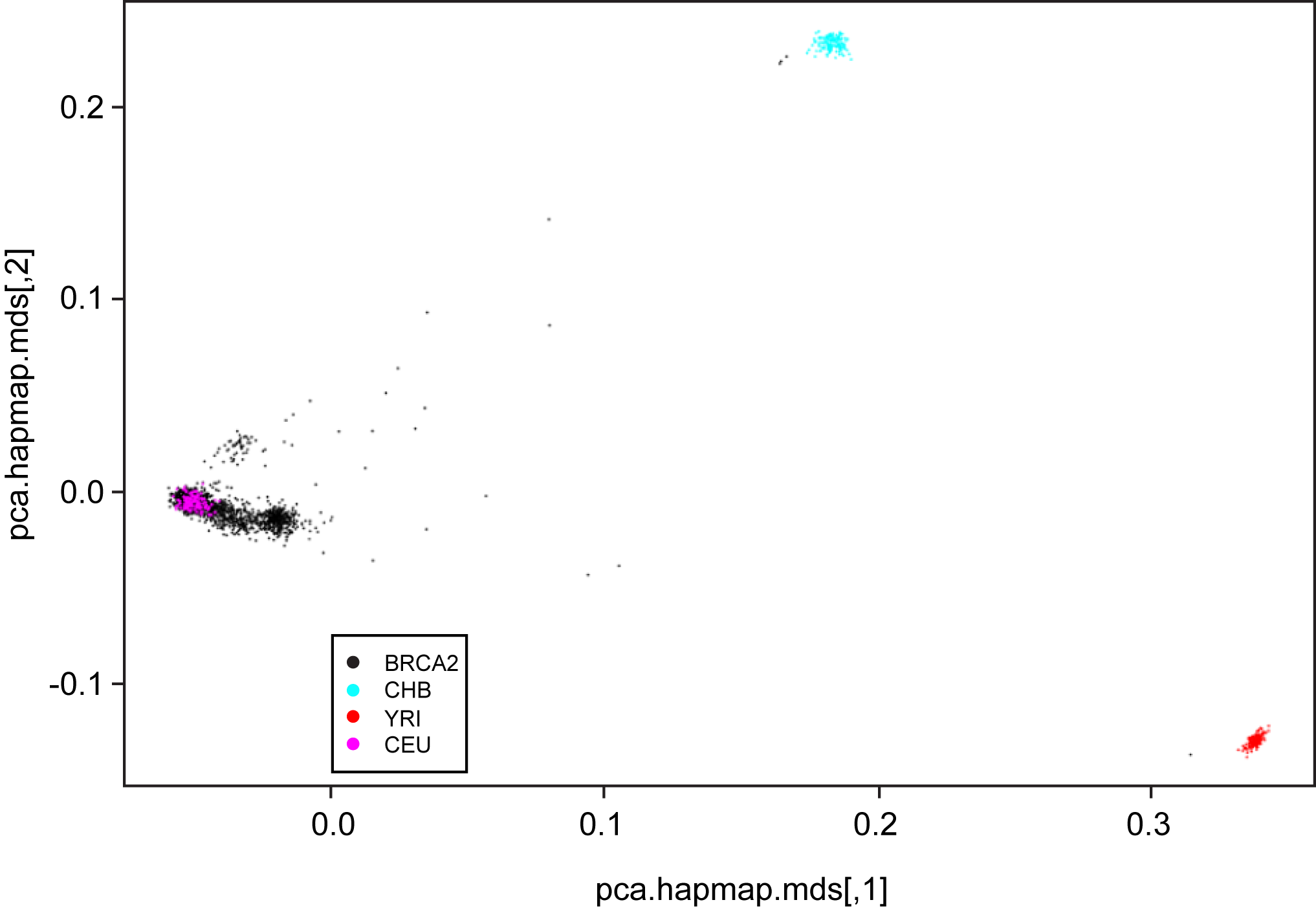

Supplement: Figure S2 — Quantile-quantile plot comparing expected distribution of chi-square values and observed chi-square values from a genome-wide scan of breast cancer cases and unaffected BRCA2 carriers. (0.31 MB TIF) [file pgen.1001183.s002.tif]

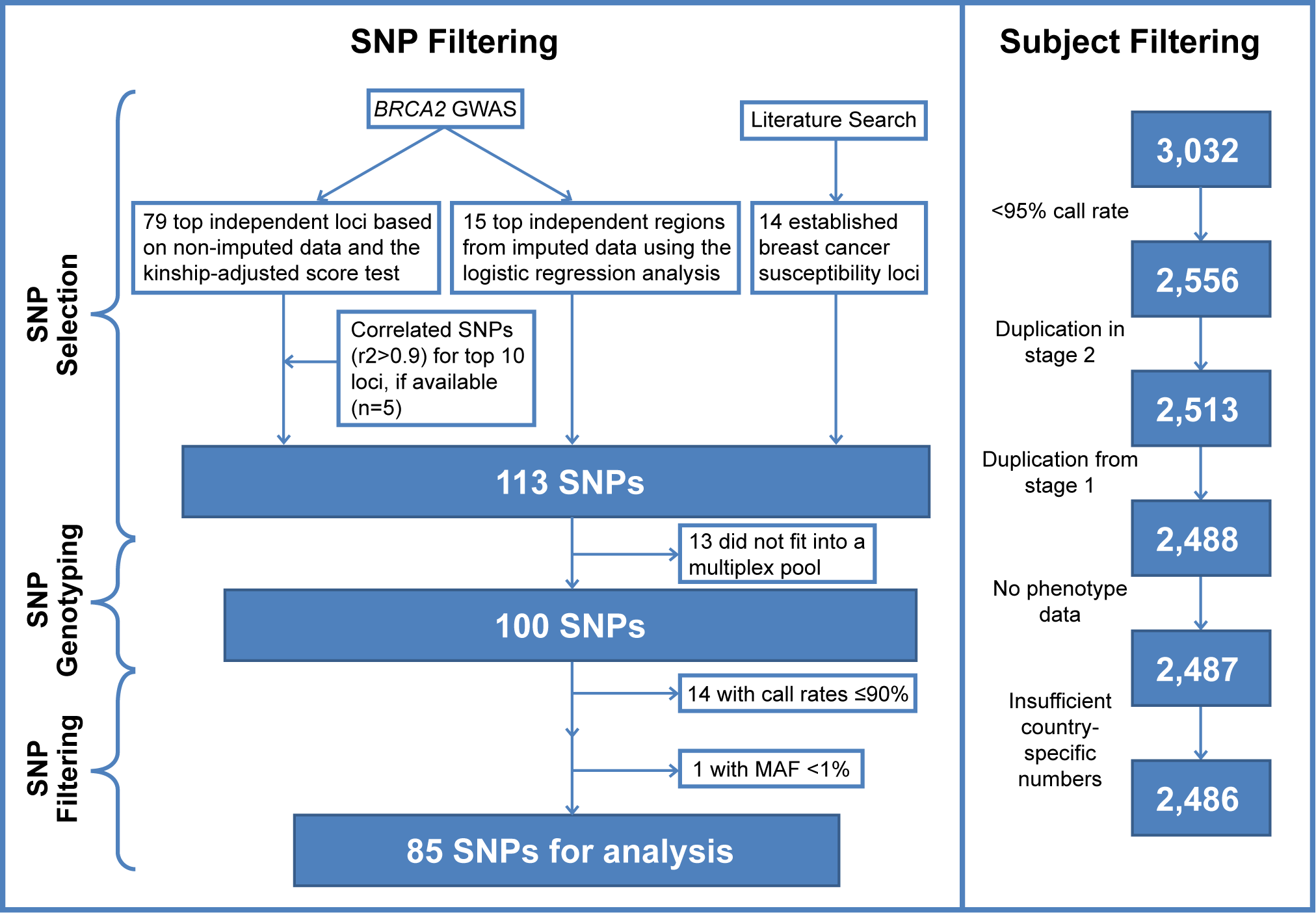

Supplement: Figure S3 — Manhattan plot of p-values by chromosomal position from a genome-wide scan of breast cancer cases and unaffected BRCA2 carriers [Visualized using SVS7 (Goldenhelix)]. (0.51 MB TIF) [file pgen.1001183.s003.tif]

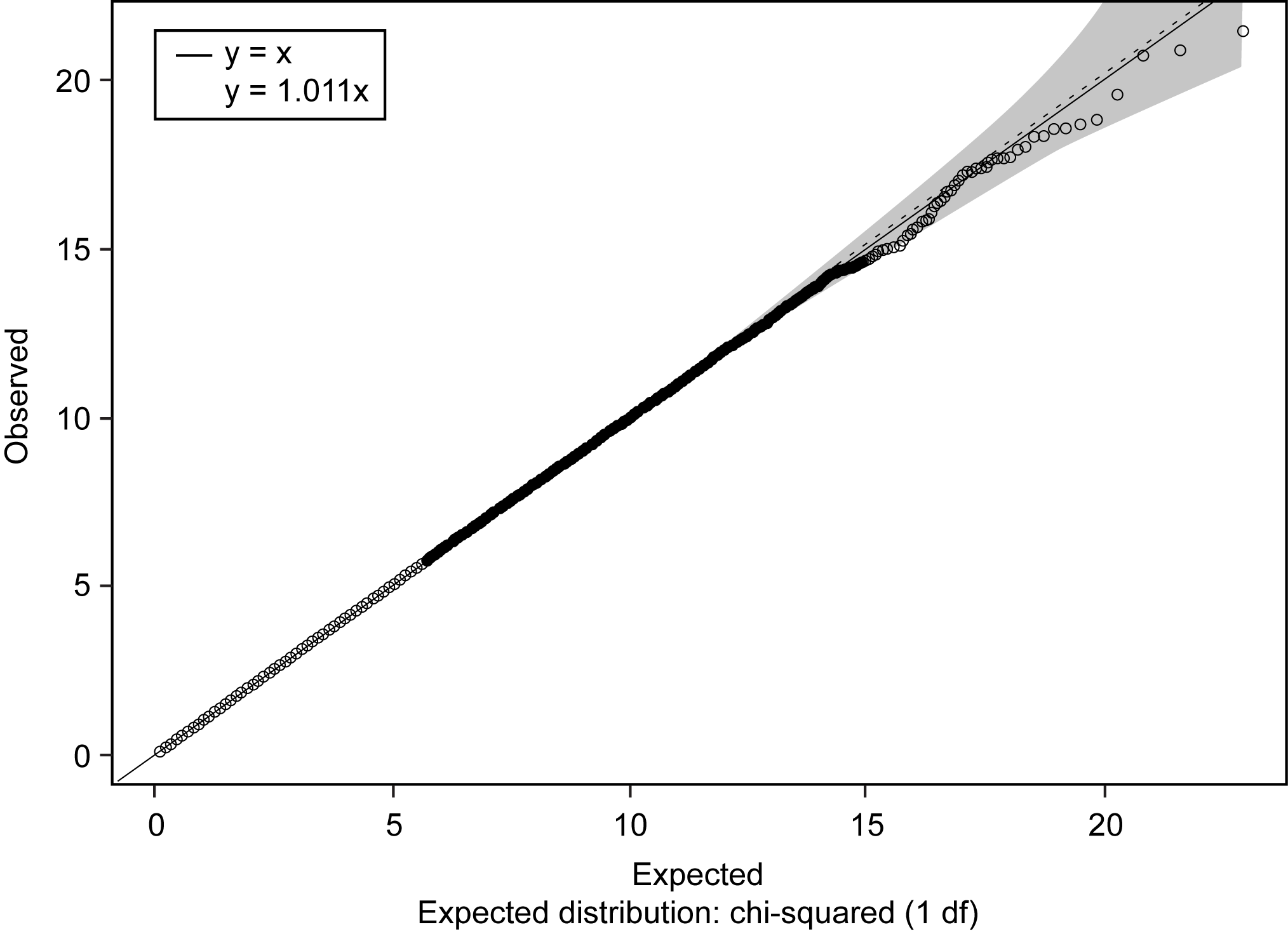

Supplement: Figure S4 — Quantile-quantile plot comparing expected distribution of p-values and observed p-values of association of common copy number polymorphisms (CNPs) from a genome-wide scan of breast cancer cases and unaffected BRCA2 carriers. (0.32 MB TIF) [file pgen.1001183.s004.tif]

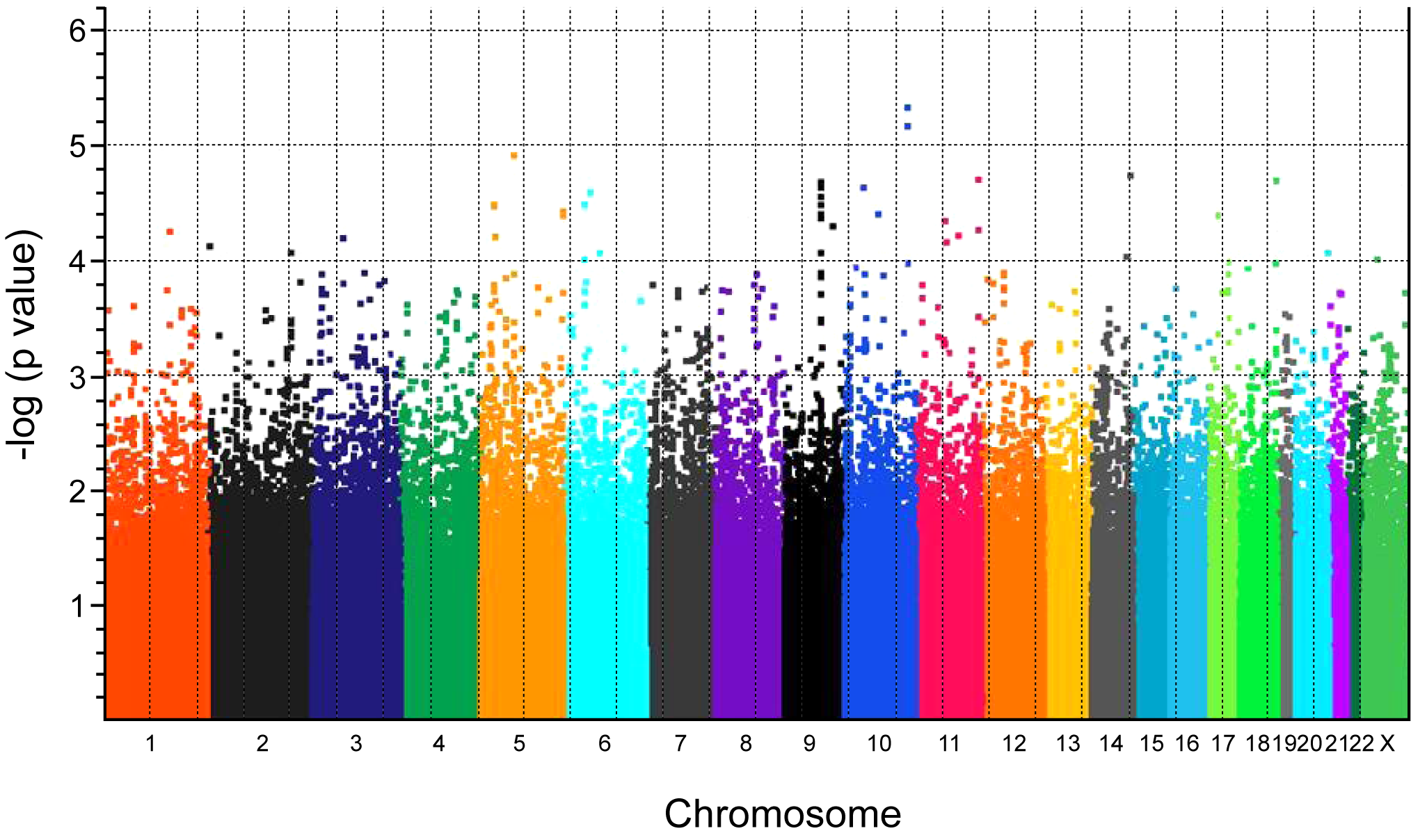

Supplement: Figure S5 — Principal components analysis, including all eligible (after filtering) BRCA2 stage 1 samples and HapMap samples. (2.39 MB TIF) [file pgen.1001183.s005.tif]

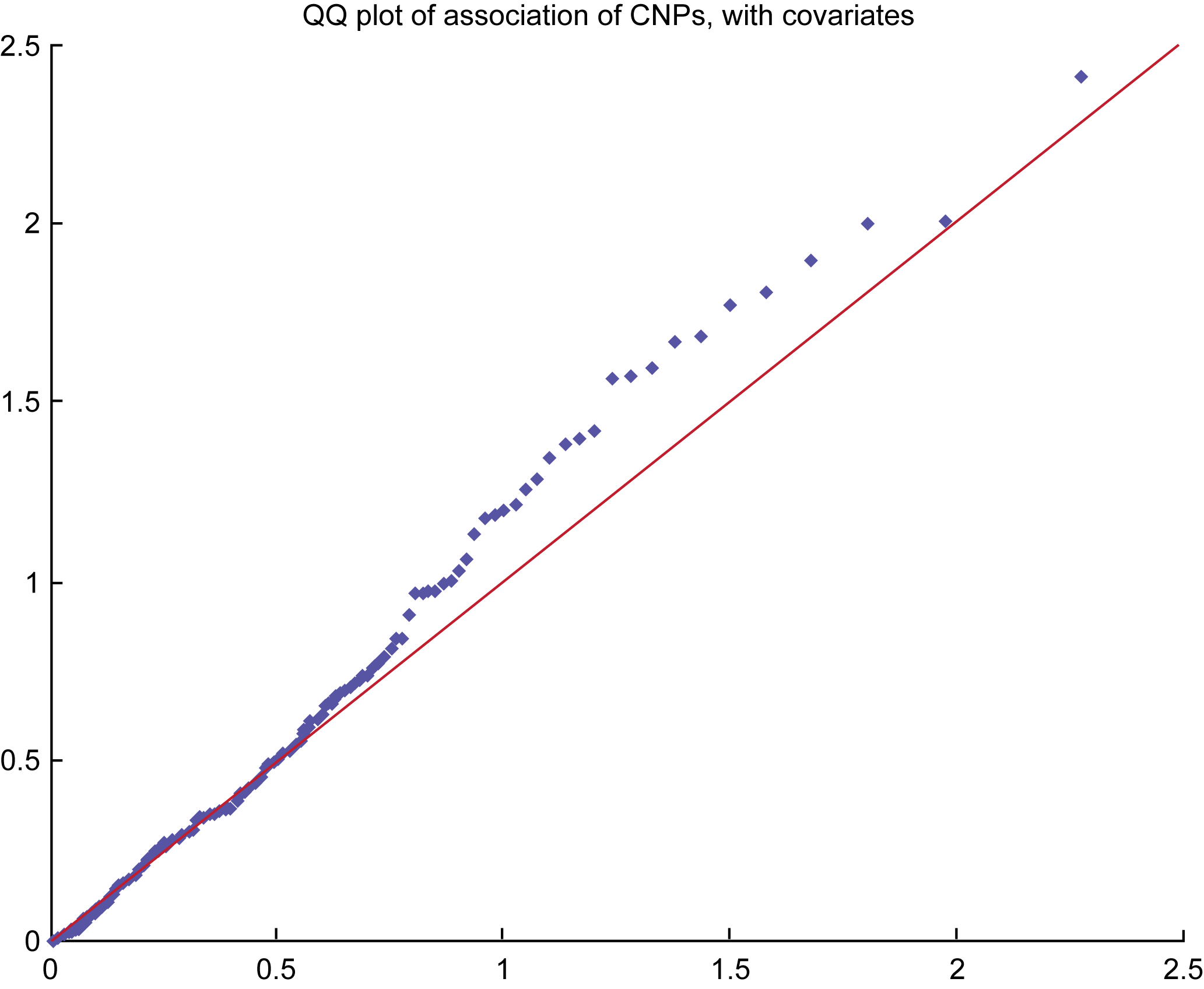

Supplement: Figure S6 — Data filtering of stage 2 BRCA2 GWAS. (0.35 MB TIF) [file pgen.1001183.s006.tif]
