# Supplementary material for: Common Genetic Variants and Modification of Penetrance of BRCA2-Associated Breast Cancer
Source: PLoS Genet. 2010 Oct 28;6(10):e1001183. doi: 10.1371/journal.pgen.1001183 (PMC2965747; doi:10.1371/journal.pgen.1001183)
Supplement: Table S1 — List of 59 BRCA interactors or regulators and their gene association p-values to breast cancer age of onset in BRCA2 mutation carriers. (0.13 MB DOC) [file pgen.1001183.s007.doc]

| **Table S1. List of 59 *BRCA* interactors or regulators and their gene association p-values to breast cancer age of onset in *BRCA2* mutation carriers** | | | | | | | | | |  | | |
| --- | --- | --- | --- | --- | --- | --- | --- | --- | --- | --- | --- | --- |
| **Gene symbol** | **Entrez ID** | **Gene association**  **p-value** | **Gene Chr #** | **Gene chr start position, bp** | **Gene chr end position, bp** | **Gene size, kb** | **# SNPs per gene** | **Best local SNP rs #** | **Best local SNP chr #** | **Best local SNP chr position, bp** | **Best local SNP z-score** | **Best local SNP p-value** |
| CHEK2 | 11200 | 0.0074 | 22 | 27413730 | 27467822 | 54 | 28 | rs7573500 | 22 | 27382951 | 3.2006 | 0.001371495 |
| FANCE | 2178 | 0.0116 | 6 | 35528115 | 35542859 | 15 | 29 | rs6816370 | 6 | 35448278 | 3.2236 | 0.001266072 |
| HIF1A | 3091 | 0.0295 | 14 | 61231871 | 61284730 | 53 | 29 | rs10864 | 14 | 61183857 | 2.9614 | 0.003062271 |
| PSMD6 | 9861 | 0.0519 | 3 | 63971270 | 63984160 | 13 | 38 | rs875294 | 3 | 63984548 | 2.9247 | 0.003448209 |
| USF2 | 7392 | 0.0838 | 19 | 40451735 | 40462558 | 11 | 38 | rs6874450 | 19 | 40498729 | 2.8918 | 0.003830369 |
| CDC45L | 8318 | 0.0937 | 22 | 17847415 | 17888135 | 41 | 32 | rs11767058 | 22 | 17793706 | 2.7067 | 0.006796346 |
| KIF4A | 24137 | 0.1000 | 23 | 69426619 | 69557461 | 131 | 45 | rs807190 | 23 | 69583640 | 2.6291 | 0.008561367 |
| XRCC2 | 7516 | 0.1311 | 7 | 151974519 | 152004183 | 30 | 26 | rs726568 | 7 | 152062784 | 2.6245 | 0.008678208 |
| AURKA | 6790 | 0.1417 | 20 | 54377851 | 54400758 | 23 | 40 | rs952579 | 20 | 54430807 | 2.7319 | 0.006297812 |
| C11orf30 | 56946 | 0.1717 | 11 | 75833716 | 75940237 | 107 | 39 | rs9998043 | 11 | 75731175 | 2.4862 | 0.012913237 |
| TP53 | 7157 | 0.1717 | 17 | 7512444 | 7531588 | 19 | 21 | rs7180600 | 17 | 7519404 | 2.3769 | 0.017457893 |
| PARP1 | 142 | 0.1940 | 1 | 224615014 | 224662424 | 47 | 56 | rs6456356 | 1 | 224590183 | 2.5599 | 0.01047171 |
| BACH1 | 571 | 0.2013 | 21 | 29593090 | 29656086 | 63 | 30 | rs6674994 | 21 | 29530655 | 2.3047 | 0.021181138 |
| FANCF | 2188 | 0.2071 | 11 | 22600654 | 22603963 | 3 | 23 | rs16867228 | 11 | 22653268 | 2.3509 | 0.018729795 |
| FANCC | 2176 | 0.2228 | 9 | 96901156 | 97119812 | 219 | 48 | rs1421872 | 9 | 97216994 | 2.4076 | 0.01605968 |
| BARD1 | 580 | 0.2328 | 2 | 215301519 | 215382673 | 81 | 56 | rs17098843 | 2 | 215289203 | 2.5692 | 0.01019362 |
| SP1 | 6667 | 0.2340 | 12 | 52060245 | 52096493 | 36 | 13 | rs17151807 | 12 | 52024481 | 2.0725 | 0.0382207 |
| FANCD2 | 2177 | 0.2483 | 3 | 10043112 | 10118614 | 76 | 30 | rs2267232 | 3 | 10128559 | 2.2246 | 0.026111335 |
| MCPH1 | 79648 | 0.2623 | 8 | 6251528 | 6493434 | 242 | 212 | rs462402 | 8 | 6425723 | 3.3122 | 0.000925627 |
| VHL | 7428 | 0.2806 | 3 | 10158318 | 10168746 | 10 | 29 | rs2267232 | 3 | 10128559 | 2.2246 | 0.026111335 |
| FANCB | 2187 | 0.2886 | 23 | 14771449 | 14801105 | 30 | 16 | rs9517558 | 23 | 14875784 | 1.9985 | 0.045658373 |
| BLM | 641 | 0.3094 | 15 | 89061582 | 89159690 | 98 | 38 | rs4141261 | 15 | 89184146 | 2.2789 | 0.022672116 |
| FHL2 | 2274 | 0.3148 | 2 | 105343714 | 105421662 | 78 | 97 | rs2322757 | 2 | 105305862 | 2.8754 | 0.004035774 |
| FANCL | 55120 | 0.3376 | 2 | 58239881 | 58322019 | 82 | 32 | rs1281404 | 2 | 58376628 | 2.0782 | 0.037695257 |
| PALB2 | 79728 | 0.3508 | 16 | 23521983 | 23560179 | 38 | 37 | rs12124983 | 16 | 23587787 | 2.2225 | 0.026247548 |
| RAD51L1 | 5890 | 0.3632 | 14 | 67356261 | 68132367 | 776 | 191 | rs879750 | 14 | 68080958 | 2.829 | 0.004668822 |
| XRCC3 | 7517 | 0.3770 | 14 | 103233706 | 103251576 | 18 | 19 | rs2531728 | 14 | 103268004 | 1.9406 | 0.052302922 |
| IGFBP2 | 3485 | 0.3988 | 2 | 217206371 | 217237403 | 31 | 40 | rs36693 | 2 | 217182678 | 2.2468 | 0.024652656 |
| ACACA | 31 | 0.4472 | 17 | 32516039 | 32841015 | 325 | 56 | rs12005581 | 17 | 32551878 | 2.119 | 0.034087417 |
| GPX1 | 2876 | 0.4528 | 3 | 49369612 | 49370795 | 1 | 8 | rs201820 | 3 | 49425868 | 1.6585 | 0.097224059 |
| MSH2 | 4436 | 0.4990 | 2 | 47483766 | 47563864 | 80 | 23 | rs9862229 | 2 | 47437468 | 1.8985 | 0.057630558 |
| MLH1 | 4292 | 0.5144 | 3 | 37009982 | 37067341 | 57 | 32 | rs9406758 | 3 | 37091046 | 1.8459 | 0.064903493 |
| IRS1 | 3667 | 0.5333 | 2 | 227304276 | 227371750 | 67 | 47 | rs12017262 | 2 | 227330075 | 2.086 | 0.0369788 |
| BCCIP | 56647 | 0.5620 | 10 | 127502093 | 127532254 | 30 | 21 | rs12522427 | 10 | 127485187 | 1.7044 | 0.088311231 |
| NFKB1 | 4790 | 0.5779 | 4 | 103641517 | 103757507 | 116 | 52 | rs6503749 | 4 | 103610299 | 1.9648 | 0.04943643 |
| HUS1 | 3364 | 0.6464 | 7 | 47970307 | 47985771 | 15 | 37 | rs457587 | 7 | 48060754 | 1.8352 | 0.06648134 |
| USF1 | 7391 | 0.6499 | 1 | 159275664 | 159282381 | 7 | 23 | rs2249405 | 1 | 159340716 | 1.6437 | 0.100247155 |
| BRIP1 | 83990 | 0.6717 | 17 | 57114766 | 57295537 | 181 | 27 | rs619351 | 17 | 57085167 | 1.5886 | 0.112153065 |
| IGFBP5 | 3488 | 0.6772 | 2 | 217245072 | 217268517 | 23 | 47 | rs8092119 | 2 | 217212357 | 2.0591 | 0.039488461 |
| RAD51 | 5888 | 0.6917 | 15 | 38774650 | 38811648 | 37 | 17 | rs11862295 | 15 | 38666270 | 1.4347 | 0.151382015 |
| PLEC1 | 5339 | 0.7037 | 8 | 145061308 | 145121531 | 60 | 9 | rs11080775 | 8 | 145109561 | 1.3949 | 0.163052278 |
| IGF1R | 3480 | 0.7160 | 15 | 97010283 | 97325282 | 315 | 112 | rs10814017 | 15 | 97343311 | 2.091 | 0.036525821 |
| UIMC1 | 51720 | 0.7252 | 5 | 176264611 | 176366049 | 101 | 36 | rs1759417 | 5 | 176396647 | 1.6131 | 0.106718049 |
| IGFBP1 | 3484 | 0.7256 | 7 | 45894483 | 45899792 | 5 | 20 | rs12421963 | 7 | 45875214 | 1.5145 | 0.129906071 |
| FANCG | 2189 | 0.7283 | 9 | 35063834 | 35070013 | 6 | 21 | rs2199471 | 9 | 35125239 | 1.5198 | 0.128551997 |
| GADD45A | 1647 | 0.7351 | 1 | 67923470 | 67926607 | 3 | 50 | rs2599675 | 1 | 67859703 | 1.8738 | 0.060962887 |
| ELF1 | 1997 | 0.7723 | 13 | 40404163 | 40454418 | 50 | 20 | rs6687920 | 13 | 40542956 | 1.3307 | 0.183293613 |
| SKP2 | 6502 | 0.7796 | 5 | 36187945 | 36219904 | 32 | 33 | rs1016392 | 5 | 36240260 | 1.516 | 0.129506912 |
| SOD2 | 6648 | 0.8113 | 6 | 160020138 | 160034343 | 14 | 34 | rs13036459 | 6 | 160124316 | 1.4863 | 0.13719896 |
| SHFM1 | 7979 | 0.8151 | 7 | 96156014 | 96177139 | 21 | 45 | rs1875845 | 7 | 96215976 | 1.7123 | 0.086841067 |
| BRCA2 | 675 | 0.8377 | 13 | 31787616 | 31871809 | 84 | 51 | rs9901510 | 13 | 31819306 | 1.6197 | 0.105291869 |
| RAD51C | 5889 | 0.8498 | 17 | 54124961 | 54166691 | 42 | 14 | rs9307626 | 17 | 54195586 | 1.1074 | 0.26812735 |
| IGF1 | 3479 | 0.8615 | 12 | 101313774 | 101398508 | 85 | 59 | rs7091181 | 12 | 101454225 | 1.5944 | 0.110843724 |
| KPNA2 | 3838 | 0.9225 | 17 | 63462309 | 63473432 | 11 | 4 | rs7603751 | 17 | 63446425 | 0.7529 | 0.451507412 |
| PSMD3 | 5709 | 0.9235 | 17 | 35390585 | 35407738 | 17 | 19 | rs12504621 | 17 | 35320546 | 0.9917 | 0.321336946 |
| ATM | 472 | 0.9253 | 11 | 107598768 | 107745036 | 146 | 32 | rs17687462 | 11 | 107491628 | 1.0785 | 0.280801826 |
| FANCM | 57697 | 0.9557 | 14 | 44674885 | 44739843 | 65 | 5 | rs4853833 | 14 | 44706257 | 0.6576 | 0.510776252 |
| DMC1 | 11144 | 0.9721 | 22 | 37244899 | 37296135 | 51 | 24 | rs2621212 | 22 | 37349799 | 0.7231 | 0.46963286 |
| ZNF350 | 59348 | 0.9725 | 19 | 57159404 | 57181891 | 22 | 23 | rs2951090 | 19 | 57151862 | 0.7851 | 0.432403116 |
