# Supplementary material for: Common Genetic Variants and Modification of Penetrance of BRCA2-Associated Breast Cancer
Source: PLoS Genet. 2010 Oct 28;6(10):e1001183. doi: 10.1371/journal.pgen.1001183 (PMC2965747; doi:10.1371/journal.pgen.1001183)
Supplement: Table S2 — Ranked results for the 85 SNPs successfully genotyped in stage 2, BRCA2 GWAS. (0.13 MB DOC) [file pgen.1001183.s008.doc]

| **Table S2. Ranked results for the 85 SNPs successfully genotyped in stage 2, BRCA2 GWAS** | | | | | | | | | | | |
| --- | --- | --- | --- | --- | --- | --- | --- | --- | --- | --- | --- |
|  |  | **Stage 1** | | | **Stage 2** | | | **Stage 1 and 2** | | | |
| **SNP Name** | **allele1,alelle2** | **N** | **Chi-square** | **Robust  p-value** | **N** | **Chi-square** | **Robust  p-value** | **N** | **HR** | **95% CI** | **Robust  p-value** |
| rs2981575 | AG | 1686 | 20.499 | 5.97E-06 | 2485 | 12.373 | 4.36E-04 | 4171 | 1.28 | 1.18-1.39 | 1.22E-08 |
| rs16917302 | AC | 1702 | 18.426 | 1.77E-05 | 2486 | 2.216 | 1.37E-01 | 4188 | 0.75 | 0.66-0.86 | 3.76E-05 |
| rs3803662 | GA | 1703 | 7.610 | 5.81E-03 | 2485 | 8.942 | 2.79E-03 | 4188 | 1.20 | 1.10-1.31 | 4.94E-05 |
| rs311499 | CT | 1674 | 17.138 | 3.48E-05 | 2464 | 2.316 | 1.28E-01 | 4138 | 0.72 | 0.61-0.85 | 6.64E-05 |
| rs3803825 | GA | 1702 | 14.352 | 1.52E-04 | 2486 | 2.493 | 1.14E-01 | 4188 | 1.24 | 1.11-1.39 | 1.18E-04 |
| rs888708 | TA | 1680 | 17.604 | 2.72E-05 | 2464 | 0.948 | 3.30E-01 | 4144 | 0.84 | 0.76-0.92 | 2.03E-04 |
| rs7937776 | TC | 1703 | 17.830 | 2.42E-05 | 2484 | 0.954 | 3.29E-01 | 4187 | 1.22 | 1.10-1.35 | 2.24E-04 |
| rs4794469 | AC | 1701 | 15.019 | 1.06E-04 | 2485 | 1.483 | 2.23E-01 | 4186 | 1.22 | 1.09-1.36 | 2.51E-04 |
| rs11254759 | GA | 1702 | 13.528 | 2.35E-04 | 2485 | 1.406 | 2.36E-01 | 4187 | 0.80 | 0.71-0.91 | 4.29E-04 |
| rs2057537 | GA | 1703 | 13.662 | 2.19E-04 | 2486 | 1.231 | 2.67E-01 | 4189 | 1.57 | 1.21-2.05 | 4.79E-04 |
| rs7895644 | TG | 1701 | 13.595 | 2.27E-04 | 2469 | 1.219 | 2.70E-01 | 4170 | 0.81 | 0.71-0.91 | 5.10E-04 |
| rs7036417 | CT | 1702 | 15.904 | 6.67E-05 | 2473 | 0.669 | 4.13E-01 | 4175 | 1.17 | 1.07-1.27 | 5.14E-04 |
| rs896617 | GA | 1701 | 13.730 | 2.11E-04 | 2391 | 1.219 | 2.70E-01 | 4092 | 0.85 | 0.77-0.93 | 5.22E-04 |
| rs6878576 | CT | 1698 | 19.218 | 1.17E-05 | 2486 | 0.165 | 6.85E-01 | 4184 | 1.24 | 1.10-1.39 | 5.38E-04 |
| rs3108049 | CT | 1693 | 14.286 | 1.57E-04 | 2485 | 0.986 | 3.21E-01 | 4178 | 0.83 | 0.75-0.92 | 6.49E-04 |
| rs9380618 | GA | 1686 | 18.183 | 2.01E-05 | 2435 | 0.172 | 6.79E-01 | 4121 | 0.87 | 0.80-0.94 | 7.64E-04 |
| rs807339 | GC | 1698 | 18.286 | 1.90E-05 | 2485 | 0.166 | 6.84E-01 | 4183 | 0.76 | 0.64-0.89 | 8.25E-04 |
| rs4483901 | GA | 1697 | 13.926 | 1.90E-04 | 2449 | 0.824 | 3.64E-01 | 4146 | 1.15 | 1.06-1.25 | 9.24E-04 |
| rs2799359 | GT | 1703 | 18.725 | 1.51E-05 | 2484 | 0.001 | 9.70E-01 | 4187 | 0.60 | 0.44-0.82 | 9.64E-04 |
| rs11254771 | AG | 1702 | 14.186 | 1.66E-04 | 2486 | 0.501 | 4.79E-01 | 4188 | 0.81 | 0.72-0.92 | 1.03E-03 |
| rs10804562 | GA | 1700 | 13.685 | 2.16E-04 | 2463 | 0.426 | 5.14E-01 | 4163 | 0.85 | 0.76-0.94 | 1.83E-03 |
| rs4672790 | TC | 1700 | 14.491 | 1.41E-04 | 2483 | 0.211 | 6.46E-01 | 4183 | 0.87 | 0.80-0.95 | 2.49E-03 |
| rs2433288 | GC | 1691 | 16.234 | 5.60E-05 | 2484 | 0.051 | 8.21E-01 | 4175 | 1.14 | 1.05-1.24 | 2.68E-03 |
| rs7317238 | TG | 1696 | 14.084 | 1.75E-04 | 2466 | 0.200 | 6.55E-01 | 4162 | 0.84 | 0.75-0.94 | 3.02E-03 |
| rs7078452 | CT | 1691 | 16.378 | 5.19E-05 | 2485 | 0.000 | 9.96E-01 | 4176 | 1.19 | 1.06-1.33 | 3.13E-03 |
| rs17701675 | GA | 1702 | 13.467 | 2.43E-04 | 2414 | 0.130 | 7.19E-01 | 4116 | 1.14 | 1.05-1.24 | 3.31E-03 |
| rs17517571 | GA | 1698 | 15.309 | 9.13E-05 | 2486 | 0.012 | 9.12E-01 | 4184 | 0.83 | 0.73-0.94 | 3.58E-03 |
| rs10906720 | CG | 1702 | 15.550 | 8.04E-05 | 2483 | 0.019 | 8.90E-01 | 4185 | 0.83 | 0.73-0.94 | 3.67E-03 |
| rs9976694 | CT | 1690 | 13.047 | 3.04E-04 | 2466 | 0.183 | 6.69E-01 | 4156 | 1.14 | 1.05-1.24 | 3.94E-03 |
| rs1319677 | AG | 1700 | 15.852 | 6.85E-05 | 2453 | 0.001 | 9.70E-01 | 4153 | 0.89 | 0.82-0.96 | 3.98E-03 |
| rs6527237 | GA | 1696 | 15.570 | 7.95E-05 | 2486 | 0.000 | 9.83E-01 | 4182 | 1.13 | 1.04-1.23 | 4.63E-03 |
| rs290274 | GA | 1702 | 15.195 | 9.69E-05 | 2485 | 0.035 | 8.52E-01 | 4187 | 0.89 | 0.82-0.97 | 6.69E-03 |
| rs998518 | TG | 1688 | 14.851 | 1.16E-04 | 2466 | 0.005 | 9.45E-01 | 4154 | 1.13 | 1.04-1.23 | 6.83E-03 |
| rs10993693 | AG | 1701 | 13.780 | 2.06E-04 | 2486 | 0.002 | 9.66E-01 | 4187 | 1.12 | 1.03-1.22 | 6.99E-03 |
| rs575536 | CT | 1703 | 14.552 | 1.36E-04 | 2477 | 0.001 | 9.76E-01 | 4180 | 1.15 | 1.04-1.27 | 7.60E-03 |
| rs1345787 | TC | 1699 | 14.179 | 1.66E-04 | 2482 | 0.001 | 9.77E-01 | 4181 | 1.12 | 1.03-1.22 | 7.76E-03 |
| rs12448959 | GC | 1703 | 14.223 | 1.62E-04 | 2486 | 0.023 | 8.80E-01 | 4189 | 1.25 | 1.07-1.47 | 8.87E-03 |
| rs2433294 | GC | 1699 | 13.960 | 1.87E-04 | 2478 | 0.040 | 8.42E-01 | 4177 | 1.14 | 1.03-1.27 | 9.95E-03 |
| rs7243090 | TA | 1702 | 16.427 | 5.06E-05 | 2481 | 0.220 | 6.39E-01 | 4183 | 0.89 | 0.82-0.97 | 1.01E-02 |
| rs2222309 | CT | 1700 | 12.006 | 5.30E-04 | 2486 | 0.012 | 9.14E-01 | 4186 | 1.17 | 1.04-1.31 | 1.23E-02 |
| rs9879234 | TC | 1702 | 13.722 | 2.12E-04 | 2479 | 0.075 | 7.84E-01 | 4181 | 0.90 | 0.82-0.98 | 1.26E-02 |
| rs11822285 | CT | 1703 | 15.899 | 6.68E-05 | 2460 | 0.552 | 4.58E-01 | 4163 | 0.90 | 0.83-0.98 | 1.56E-02 |
| rs786990 | GA | 1664 | 15.529 | 8.12E-05 | 2483 | 0.378 | 5.39E-01 | 4147 | 0.90 | 0.83-0.98 | 1.74E-02 |
| rs1240951 | GA | 1703 | 11.022 | 9.01E-04 | 2486 | 0.002 | 9.60E-01 | 4189 | 0.87 | 0.77-0.98 | 1.82E-02 |
| rs12553524 | GT | 1696 | 13.918 | 1.91E-04 | 2486 | 0.311 | 5.77E-01 | 4182 | 0.90 | 0.82-0.98 | 2.03E-02 |
| rs2053741 | GA | 1703 | 12.421 | 4.25E-04 | 2482 | 0.142 | 7.06E-01 | 4185 | 0.87 | 0.77-0.97 | 2.10E-02 |
| rs248132 | TA | 1680 | 10.194 | 1.41E-03 | 2480 | 0.045 | 8.32E-01 | 4160 | 0.88 | 0.77-0.99 | 3.05E-02 |
| rs12304191 | GA | 1702 | 14.019 | 1.81E-04 | 2482 | 0.669 | 4.13E-01 | 4184 | 0.85 | 0.73-0.98 | 3.34E-02 |
| rs3817198 | TC | 1684 | 2.902 | 8.85E-02 | 2482 | 1.432 | 2.31E-01 | 4166 | 1.09 | 1.00-1.19 | 4.00E-02 |
| rs13071097 | AC | 1668 | 12.558 | 3.94E-04 | 2484 | 0.550 | 4.58E-01 | 4152 | 0.92 | 0.84-1.00 | 4.48E-02 |
| rs12217435 | CG | 1702 | 14.447 | 1.44E-04 | 2485 | 1.146 | 2.84E-01 | 4187 | 1.18 | 1.01-1.38 | 4.58E-02 |
| rs2075976 | AC | 1694 | 11.765 | 6.03E-04 | 2486 | 0.696 | 4.04E-01 | 4180 | 1.17 | 1.01-1.37 | 5.65E-02 |
| rs17070289 | TG | 1699 | 14.487 | 1.41E-04 | 2486 | 1.702 | 1.92E-01 | 4185 | 1.24 | 0.98-1.57 | 7.94E-02 |
| rs11150978 | AG | 1700 | 21.459 | 3.62E-06 | 2483 | 6.000 | 1.43E-02 | 4183 | 0.91 | 0.81-1.01 | 8.93E-02 |
| rs11660857 | CT | 1696 | 19.454 | 1.03E-05 | 2485 | 5.601 | 1.79E-02 | 4181 | 0.91 | 0.82-1.02 | 1.08E-01 |
| rs6821660 | AC | 1701 | 14.422 | 1.46E-04 | 2484 | 2.535 | 1.11E-01 | 4185 | 1.18 | 0.98-1.41 | 1.10E-01 |
| rs7116050 | GC | 1703 | 19.346 | 1.09E-05 | 2486 | 5.388 | 2.03E-02 | 4189 | 0.90 | 0.80-1.03 | 1.10E-01 |
| rs7127732 | CT | 1696 | 18.174 | 2.02E-05 | 2486 | 5.506 | 1.90E-02 | 4182 | 0.91 | 0.80-1.03 | 1.30E-01 |
| rs908351 | TC | 1703 | 12.041 | 5.20E-04 | 2481 | 1.818 | 1.78E-01 | 4184 | 0.85 | 0.70-1.03 | 1.32E-01 |
| rs13257749 | CA | 1702 | 15.194 | 9.70E-05 | 2484 | 5.819 | 1.59E-02 | 4186 | 0.92 | 0.79-1.06 | 2.19E-01 |
| rs3020314 | TC | 1702 | 0.518 | 4.72E-01 | 2486 | 0.525 | 4.69E-01 | 4188 | 0.95 | 0.87-1.04 | 3.07E-01 |
| rs2180341 | AG | 1688 | 0.190 | 6.63E-01 | 2482 | 2.844 | 9.17E-02 | 4170 | 1.04 | 0.95-1.15 | 3.72E-01 |
| rs1026411 | GA | 1702 | 12.570 | 3.92E-04 | 2484 | 6.049 | 1.39E-02 | 4186 | 0.96 | 0.88-1.05 | 4.20E-01 |
| rs11750845 | TC | 1700 | 1.352 | 2.45E-01 | 2486 | 0.977 | 3.23E-01 | 4186 | 1.00 | 0.92-1.08 | 8.87E-01 |
